# p62/SQSTM1/Sequestosome-1 is an N-recognin of the N-end rule pathway which modulates autophagosome biogenesis

**DOI:** 10.1038/s41467-017-00085-7

**Published:** 2017-07-24

**Authors:** Hyunjoo Cha-Molstad, Ji Eun Yu, Zhiwei Feng, Su Hyun Lee, Jung Gi Kim, Peng Yang, Bitnara Han, Ki Woon Sung, Young Dong Yoo, Joonsung Hwang, Terry McGuire, Sang Mi Shim, Hyun Dong Song, Srinivasrao Ganipisetti, Nuozhou Wang, Jun Min Jang, Min Jae Lee, Seung Jun Kim, Kyung Ho Lee, Jin Tae Hong, Aaron Ciechanover, Inhee Mook-Jung, Kwang Pyo Kim, Xiang-Qun Xie, Yong Tae Kwon, Bo Yeon Kim

**Affiliations:** 1 World Class Institute, Anticancer Agents Research Center, Korea Research Institute of Bioscience and Biotechnology, Ochang, Cheongwon 28116 Korea; 2 Department of Drug Discovery and Development, College of Pharmacy, Chungbuk National University, Chungbuk, Cheongju 28644 Korea; 3 Department of Pharmaceutical Sciences and Computational Chemical Genomics Screening Center, School of Pharmacy, University of Pittsburgh, Pittsburgh, PA 15260 USA; 4 NIDA National Center of Excellence for Computational Drug Abuse Research, University of Pittsburgh, Pittsburgh, PA 15260 USA; 5 Drug Discovery Institute, University of Pittsburgh, Pittsburgh, PA 15260 USA; 6 Departments of Computational Biology and Structural Biology, School of Medicine, University of Pittsburgh, Pittsburgh, PA 15260 USA; 7 Protein Metabolism Medical Research Center and Department of Biomedical Sciences, College of Medicine, Seoul National University, Seoul, 03080 Korea; 8 Department of Biomolecular Science, University of Science and Technology (UST), Daejeon, 34113 South Korea; 9 Department of Applied Chemistry, College of Applied Science, Kyung Hee University, Yongin, 17104 Korea; 10 Department of Biochemistry and Biomedical Science, Seoul National University College of Medicine, Seoul, 03080 Republic of Korea; 11 Department of Biochemistry and Molecular Biology, Department of Biomedical Sciences, Seoul National University College of Medicine, Seoul, 03080 Republic of Korea; 12 Disease Target Structure Research Center, Korea Research Institute of Bioscience and Biotechnology, Daejeon, 34141 Korea; 13 Technion Integrated Cancer Center (TICC), Faculty of Medicine, Technion-Israel Institute of Technology, Haifa, 31096 Israel; 14 Ischemic/Hypoxic Disease Institute, College of Medicine, Seoul National University, Seoul, 03080 Korea; 15 QC Team Operation Part Sample Managements, Samsung Biologics, Yeonsu-gu, Incheon 21987 Korea

## Abstract

Macroautophagy mediates the selective degradation of proteins and non-proteinaceous cellular constituents. Here, we show that the N-end rule pathway modulates macroautophagy. In this mechanism, the autophagic adapter p62/SQSTM1/Sequestosome-1 is an N-recognin that binds type-1 and type-2 N-terminal degrons (N-degrons), including arginine (Nt-Arg). Both types of N-degrons bind its ZZ domain. By employing three-dimensional modeling, we developed synthetic ligands to p62 ZZ domain. The binding of Nt-Arg and synthetic ligands to ZZ domain facilitates disulfide bond-linked aggregation of p62 and p62 interaction with LC3, leading to the delivery of p62 and its cargoes to the autophagosome. Upon binding to its ligand, p62 acts as a modulator of macroautophagy, inducing autophagosome biogenesis. Through these dual functions, cells can activate p62 and induce selective autophagy upon the accumulation of autophagic cargoes. We also propose that p62 mediates the crosstalk between the ubiquitin-proteasome system and autophagy through its binding Nt-Arg and other N-degrons.

## Abstract (web summary)

Soluble misfolded proteins that fail to be degraded by the ubiquitin proteasome system (UPS) are redirected to autophagy via specific adaptors, such as p62. Here the authors show that p62 recognises N-degrons in these proteins, acting as a N-recognin from the proteolytic N-end rule pathway, and targets these cargos to autophagosomal degradation.

## Introduction

Macroautophagy generates energy and amino acids during nutrient deprivation. Because of its nature, it has long been thought an unselective process for bulk degradation of long-lived proteins and organelles. However, recent studies revealed significant selectivity in autophagic degradation of various cellular constituents, ranging from misfolded proteins and their aggregates^1, 2^ to organelles (e.g., peroxisomes^3^ and mitochondria) and invading pathogens (e.g., viruses^4^ and bacteria^5^). In most of these autophagic processes, p62 acts as a key adapter molecule that links cargoes to the autophagosome, yet little is known about the regulation of p62 and p62-dependent autophagic processes.

Approximately 30% of newly synthesized polypeptides are incorrectly folded^6^. Functional proteins may also lose their folding through post-translational conjugation (e.g., hyperphosphorylated tau in Alzheimer’s disease), endoproteolytic cleavage (e.g., amyloid β^7^), and genetic mutations (e.g., huntingtin in Huntington’s disease (HD)^8^, or various stresses^9^). The removal of these misfolded proteins requires timely cooperation between the ubiquitin-proteasome system (UPS) and macroautophagy^2, 10–12^. The majority of soluble misfolded proteins are first degraded by the UPS. However, if the UPS fails to remove misfolded proteins either due to their aggregation-prone nature or reduced proteasomal capacity, the Ub-tagged substrates are redirected to autophagy via specific adapters, such as p62^9, 13, 14^. Cargo-loaded p62 undergoes self-polymerization and is delivered to autophagosomes through its interaction with LC3, leading to lysosomal proteolysis^15, 16^. Whereas extensive studies for the past three decades elucidated fairly detailed mechanisms underlying proteolysis by the UPS, autophagic proteolysis began to receive attention in the field only recently. As such, the mechanisms underlying its regulation and spatiotemporal specificity remain poorly understood. Urgent questions in autophagic proteolysis include how p62 normally does not interfere with the UPS and is activated only when its cargoes accumulates, how the formation of cargo–p62 complexes/aggregates is synchronized with autophagic activation, and how p62-dependent autophagic proteolysis cross-talks with the UPS under various stresses.

Substrate selectivity in the UPS is determined by the timely generation of degrons on substrates, such as N-degrons^17–20^, phospho-degrons^21^, hydroxy degrons^22^, and hydrophobic degrons. The N-end rule pathway is a proteolytic pathway, in which single N-terminal residues function as N-degrons^17, 19, 23–26^. N-degrons can be directly exposed by proteolytic cleavage or generated through post-translational modifications of N-terminally exposed residues, such as N-terminal arginylation (Nt-arginylation) by *ATE1*-encoded Arg-tRNA transferases (R-transferases; EC 2.3.2)^27–29^. In mammals, N-terminal degradation determinants include positively charged (Arg, Lys, His; type-1) and bulky hydrophobic (Phe, Trp, Tyr, Leu, and Ile; type-2) residues^30–32^. These degrons are recognized by N-recognins, which mediate ubiquitination and degradation through the proteasome^33^. Known mammalian N-recognins include UBR1/E3α, UBR2, UBR4/p600, and UBR5/EDD which are characterized by the UBR box^18, 20, 34, 35^. The 70-residue zinc-finger motif of UBR1 recognizes and binds type-1 residues, including Nt-Arg, with *K*_D_ of 1.6–3.2 μM^35^. The founding N-recognins UBR1 and UBR2 use a structurally distinct N-degron-recognition domain, called the N-domain, to recognize type-2 residue^35, 36^. The N-end rule pathway has been implicated in Ub-dependent proteolysis of cytosolic proteins in various processes^23, 29, 33, 37–40^. We recently found that a set of endoplasmic reticulum (ER)-residing proteins, such as BiP/GRP78, calreticulin, and protein disulfide isomerase, are Nt-arginylated under proteasomal inhibition^11^. Arginylated BiP accumulated in the cytosol and was delivered to autophagosomes via the interaction of its Nt-Arg to p62^11^.

In this study, we show that p62 is an N-recognin that binds Nt-Arg and other N-degrons. Upon binding to Nt-Arg and synthetic ligands to its ZZ domain, p62 undergoes disulfide bond-linked self-aggregation and interacts with LC3 on autophagic membranes, leading to the co-delivery of p62 and its cargoes to the autophagosome. In addition, ligand-bound p62 induces autophagosome biogenesis. Through this dual regulation, cells can synchronize the accumulation of autophagic cargoes to p62 activation and autophagosome biogenesis in a timely fashion. Our results suggest that p62 is a key molecule in the crosstalk between the UPS and autophagy.

## Results

### p62 binds N-end rule destabilizing N-terminal residues

**Fig. 1:**
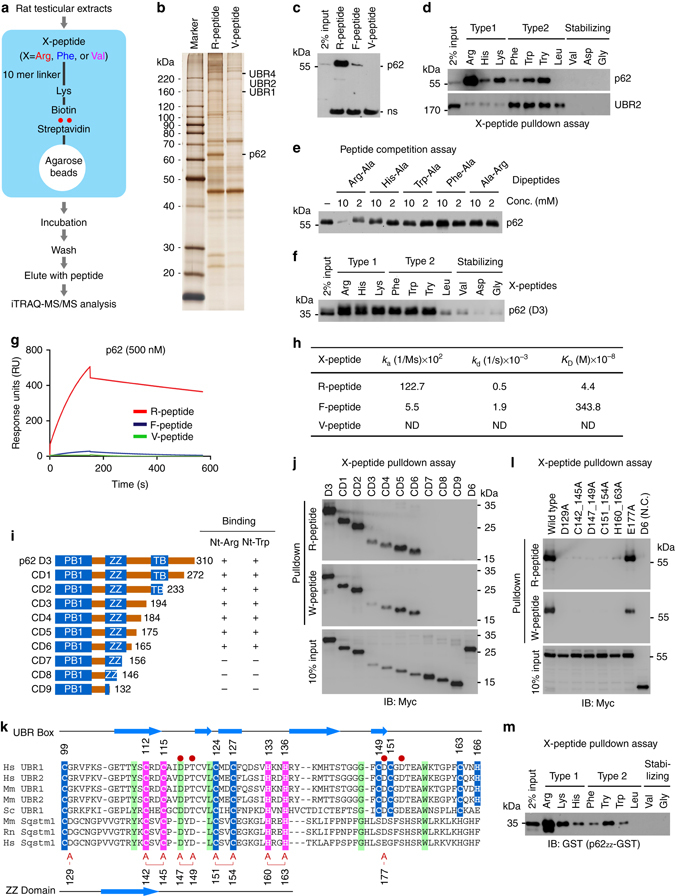
Proteomic identification of p62 as an N-recognin. **a** Schematic representation illustrating the affinity-based proteomic identification of proteins that bind to N-terminal destabilizing residues of synthetic peptides. N-terminal residues of bead-conjugated peptide are indicated by three-letter abbreviations. Biotinylated 11-mer peptides were covalently linked to streptavidine agarose beads. The pulled-down proteins were identified by iTRAQ-MS/MS analysis. **b** Silver staining of testicular proteins immobilized by 11-mer X-peptides. **c**, **d** In vitro peptide pulldown assays of endogenous p62 and UBR2 using HEK293 cells and X-peptides indicated in the figures. **e** Peptide competition assay using in vitro transcribed and translated p62. **f** X-peptide pulldown assay using p62-D3 (#1–131) ectopically expressed in HEK293 cells. **g** Biacore sensorgrams showing the kinetic analysis of p62-D3 binding to Arg-peptide, Phe-peptide or Val-peptide. **h** Surface plasmon resonance assays measuring the binding of p62 (D3) to X-peptides. Association rate constants *k*_a_, dissociation rate constants *k*_d_, equilibrium constants *K*_D_ = *k*_a_/*k*_d_. **i** Schematic representation of C-terminal deletion mutants of p62-D3 construct. **j** X-peptide pulldown assay with constructs shown in **i** where X is either Arg or Trp. **k** Alignment of the UBR boxes of UBR1 and UBR2 in comparison with p62 ZZ domain. C2H2 zinc fingers are highlighted in *pink*, and atypical binuclear zinc fingers are highlighted in *blue*. Identical residues are highlighted in *green*, and *red circles* mark residues that are essential for the recognition of destabilizing N-terminal residues. Residues of the ZZ domain that are mutated to alanine are indicated by the letter A (*red*). **l** The inhibitory effect of ZZ point mutations on p62 binding to X-peptide (X=R or W). X-peptide pulldown assay with ZZ point mutants shown in **k**. **m** X-peptide pulldown assay using 93-residue p62_ZZ_-GST containing intact ZZ domain

To identify additional N-recognins, we partially purified rat testicular proteins that bind to N-terminal residues of 11-mer X-peptides (X=Arg, Phe or Val) as affinity baits (Fig. 1a). Protein extracts were subjected to ultracentrifugation, ultrafiltration (to remove very small proteins), and chromatographic separation using X-peptide-beads. Bound proteins were eluted with Arg-Ala (for R-peptide) or Phe-Ala (for F-peptide) and analyzed using mass spectrometry (MS) (Fig. 1b; Supplementary Fig. 1a, b). The type-2 F-peptide pulled down UBR1, UBR2, and UBR4 which are known to bind type-2 residues (Supplementary Fig. 1a, c). By contrast, the type-1 R-peptide pulled down known N-recognins (UBR1, UBR4, and UBR5/EDD) as well as the autophagic adapter p62 (Fig. 1b, c; Supplementary Fig. 1d). As p62 is an adapter for misfolded proteins destined to autophagic proteolysis, we further characterized the biochemical and physiological meaning underlying the binding of testicular p62 to Nt-Arg of a synthetic peptide.

Although p62 lacks a known ubiquitination domain, it contains the UBA (Ub association) domain and, thus, has been suggested to act as a receptor that delivers ubiquitinated proteins to the proteasome^41^. To determine whether p62 is required for Ub-dependent degradation of N-end rule substrates, we monitored the metabolic stability of X-nsP4 (X=Arg or Tyr), a model N-end rule substrate derived from the Sindbis virus RNA polymerase nsP4 that bears the type-2 N-degron Nt-Tyr^31^. X-nsP4 is known to be ubiquitinated by a set of N-recognins, such as UBR1, in a manner depending on its N-degron and rapidly degraded by the proteasome^18^. Newly translated X-nsP4 was labeled/pulsed in vivo with ^35^S-Met/Cys, and its decay was monitored/chased using immunoprecipitation, followed by autoradiogram of ^35^S-Met/Cys-labeled X-nsP4. This pulse chase analysis showed that the degradation of X-nsP4 (X=Arg or Tyr) was not significantly affected by p62 knockdown (Supplementary Fig. 1e, f). As an alternative assay, we performed cycloheximide degradation assay of RGS4, a G-protein activating protein that modulates the activity of Gα subunit. We have previously shown that RGS4 is Nt-arginylated at the Cys2 residue by ATE1 R-transferase, ubiquitinated by UBR box N-recognins, and degraded by the proteasome^25^. The proteasomal degradation of RGS4 was also not significantly affected by p62 loss (Supplementary Fig. 1g). The UPS-dependent degradation of RGS4 and R-nsP4 was further validated using cells deficient in ATG5 (Supplementary Fig. 1h–j) or treated with the autophagy blocker bafilomycin A1 (Supplementary Fig. 1k). These results suggest that p62 is not essential for the turnover of N-end rule substrates through the UPS.

We next examined the binding specificity and affinity of p62 to type-1 and type-2 residues. A series of pulldown assays showed that p62 specifically bound all positively charged residues (Arg, Lys, and His; type 1) and the aromatic hydrophobic subset (Phe, Trp, and Tyr) of type-2 residues (Fig. 1d, f, h). By contrast, p62 showed no significant affinity to Nt-Leu, a β-branched hydrophobic residue that can function as a type-2 N-degron (Fig. 1d, f). The binding of testicular p62 to Nt-Arg was competitively inhibited by Arg-Ala but not Trp-Ala (Fig. 1e; lanes 1 and 2), indicative of a specific interaction to Nt-Arg. Of note, up to 50% of rat testicular p62 was immobilized to R-peptide (Fig. 1c, d; Supplementary Fig. 1d), as compared with 2–3% for UBR1, UBR4, and UBR5 (Supplementary Fig. 1c). Consistently, Biacore assay showed that p62 D3-GST fragment (residues #1–310) (Supplementary Fig. 1l) bound Nt-Arg with a *K*_D_ of 44 nM (Fig. 1g, h; Supplementary Fig. 1m), suggesting that Nt-arginylated proteins and p62 may form stable complexes. The affinity of p62 to Nt-Arg is 50–80 fold higher than that of UBR1 (*K*_D_, 1.6–3.2 μM)^35, 42^, whose binding to N-degrons is readily reversible for high processivity during the substrate recognition-ubiquitination cycle. In addition to Nt-Arg, p62 D3-GST also bound the type-2 Nt-Phe residue with a significant affinity (*K*_D_, 3.4 μM) (Fig. 1g, h; Supplementary Fig. 1m). These results suggest that p62, a well characterized autophagic adapter, binds type-1 and type-2 destabilizing N-terminal residues and may function as an N-recognin.

### p62 ZZ domain is equivalent to the UBR box of N-recognins

UBR1 recognizes type-1 and type-2 residues using its UBR box and N-domain, respectively^35^. To define the binding of p62 with N-degrons, we performed a series of X-peptide pulldown assays using 23 serially deleted p62 fragments (D1–D8, ND1–ND6, and CD1–CD9) (Supplementary Fig. 2a, c; Fig. 1i). The results collectively showed that both type-1 and type-2 residues bind to p62 through the same 46-residue region corresponding to its ZZ domain (Fig. 1j; Supplementary Fig. 2b, d, e). Sequence analysis revealed that the ZZ domain and UBR box share several structural features, including a C2H2 zinc finger motif and a few conserved residues (C128, Y140, and L150 in human p62) (Fig. 1k). Moreover, D118 in human UBR1, which forms hydrogen bonding with Nt-Arg during the N-end rule interaction^35, 43, 44^, was conserved in p62 as D147. Point mutations of zinc-coordinating or conserved residues within the 46-residue ZZ domain disrupted p62 binding to type-1 as well as type-2 degrons (Fig. 1l). Moreover, p62_ZZ_-GST, a 93-residue fragment (residue #83–175) containing only ZZ domain, retained the ability to bind type-1 and type-2 residues in a pattern identical to that of full-length p62 (Fig. 1m). These results suggest that p62 ZZ domain is a structural and functional homolog of the UBR box of N-recognins and that Nt-Arg interacts with p62 ZZ domain through the N-end rule interaction.

### Nt-Arg binding induces disulfide bond-linked p62 activation

**Fig. 2:**
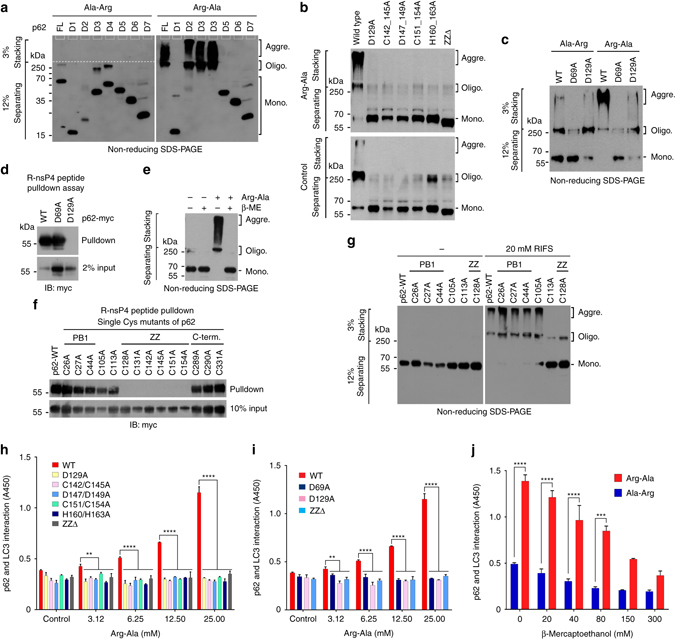
The binding of Nt-Arg to p62 ZZ domain facilitates disulfide bond-linked aggregation of p62 and p62–LC3 interaction. **a** In vitro oligomerization assay using myc/His tagged p62 deletion mutants (Supplementary Fig. 2a), followed by non-reducing SDS-PAGE and immunoblotting using a mixture of antibodies to p62 and Myc. **b** In vitro oligomerization assay using myc/His tagged wild type p62 and mutants carrying point mutations in ZZ domain. **c** In vitro p62 oligomerization assay using p62 D69A and D129A mutants in comparison with wild-type p62. **d** R-nsP4 pulldown assay using constructs used in **c**. **e** In vitro oligomerization assay of p62 ectopically expressed in HEK293 cells treated with 50 mM Arg-Ala in the presence of 50 mM β-mercaptoethanol (β-ME). **f** R-nsP4 peptide binding assay of p62 Cys mutants. Each Cys/Ala p62 mutant with myc/His tag was expressed in HEK293 cells, and 50 μg of total protein was used for pulldown. **g** In vitro oligomerization assay of p62 Cys mutants used in **f**. Cell lysates with ectopically expressed wild-type p62 and Cys mutants that can bind Nt-Arg were incubated with 20 mM RIFS tetrapeptide for 1.5 h at room temperature. **h** ELISA measuring the interaction of p62 ZZ domain mutants with LC3. Cell lysates overexpressing p62 proteins were incubated with Arg-Ala at different concentrations to allow p62 binding to LC3-GST linked to glutathione coated plates. Shown are the means (±S.D.) of three independent experiments, each performed in triplicate. A one-way ANOVA was performed to determine statistical significance (**P < 0.01; ****P < 0.0001). **i** ELISA measuring p62_D69A_ interaction with LC3 as described in **h**. The graph represents the mean (±S.D.) of three independent experiments. Statistical significance was calculated using a one-way ANOVA test (**P < 0.01; ****P < 0.0001). **j** Similar to **i** except that p62–LC3 interaction was measured in the presence of 25 mM Arg-Ala or Ala-Arg in combination with β-mercaptoethanol (β-ME) at concentrations indicated. Data represent the mean (±S.D.) of three independent experiments. Statistical significance was determined using a one-way ANOVA test (***P < 0.001; ****P < 0.0001)

We have previously observed that the addition of the dipeptide Arg-Ala to HEK293 cell extracts enhanced p62 aggregation and interaction with LC3^11^. To further characterize the role of Nt-Arg in p62 activation, we added Arg-Ala to HEK293 cell extracts expressing p62. Non-reducing sodium dodecyl sulfate-polyacrylamide gel electrophoresis (SDS-PAGE) showed that Arg-Ala, but not Ala-Arg, induced the formation of p62 aggregates that barely migrate through 3% stacking gel and trapped just below the loading well even in the presence of SDS (Figs 2a, right panel, lane 1). Analogous assays using various p62 mutant fragments (D1–D7; Supplementary Fig. 2a and 9) showed that Arg-Ala induced the aggregation of p62 fragments containing PB1 and ZZ domains (Fig. 2a; see D2–D4 in Supplementary Fig. 2a). By contrast, D1, D5, D6, and D7 fragments lacking PB1 or/and ZZ domains (Supplementary Fig. 2a) were insensitive to Arg-Ala (Fig. 2a). Full-length p62 mutants carrying point mutations in ZZ domain that cannot interact with Nt-Arg also failed to undergo polymerization in response to Arg-Ala (Fig. 2b, top). These results suggest that the interaction of Nt-Arg to ZZ domain induces the formation of high order aggregates of p62.

The PB1 domain of p62 is known to mediate self-oligomerization through the PB1–PB1 electrostatic interaction and, thus, co-aggregation with cargoes^45^, providing a critical cargo-condensation step during autophagic proteolysis. To determine the role of the PB1 domain in ZZ domain-driven p62 aggregation, we performed X-peptide pulldown and aggregation assays using a PB1 domain mutant (D69A) lacking PB1-mediated self-polymerization activity^46^. The PB1 mutant normally bound Nt-Arg (Fig. 2d) but failed to undergo self-polymerization (Fig. 2c; lanes 4 vs. 5). These results suggest that PB1 domain is still needed when the binding of Nt-Arg to ZZ domain induces p62 aggregates.

Human p62 contains 14 Cys residues, amongst which 11 are present in PB1 or ZZ domain, indicating a possible role of disulfide bond in Nt-Arg-induced p62 polymerization. Indeed, β-mercaptoethanol, a reducing agent, caused p62 species to be resolved into monomers (Fig. 2e), suggesting that disulfide bond links p62 molecules into aggregates. To delineate the mechanism underlying disulfide bond-mediated p62 polymerization induced by Nt-Arg, each Cys residue was substituted to alanine (Ala). All Cys/Ala mutants except those whose Cys is present within ZZ domain showed normal binding to Nt-Arg (Fig. 2f). Among single Cys/Ala mutants that retain the ability to bind Nt-Arg, only the mutant of Cys113, which is located in the loop between PB1 and ZZ domain, lost its ability to polymerize in response to Nt-Arg (Fig. 2g; Supplementary Fig. 3a). This suggests that Cys113 is involved in inter-disulfide bond formation during Nt-Arg induced p62 polymerization. Consistently, liquid chromatography (LC)–MS/MS analysis of oligomeric p62 species showed that Cys113 participates in disulfide bonding during polymerization (Supplementary Fig. 3b, c).

Cargo-loaded p62 aggregates are delivered to the autophagosome through interaction with LC3 on the autophagosomal membrane. GST-pulldown assays coupled with enzyme-linked immunosorbent assay (ELISA) showed that the addition of Arg-Ala to HEK293 extracts enhanced p62 interaction with LC3 (Fig. 2h). A ZZ-deleted p62 mutant did not respond to Arg-Ala (Fig. 2h). Moreover, full-length p62 proteins with point mutations, which cannot bind Nt-Arg, were invariably insensitive to Nt-Arg in LC3 interaction (Fig. 2h). The PB1 domain mutant (D69A) failed to respond to Arg-Ala (Fig. 2i), indicating that p62 may interact with LC3 as an oligomer. The enhanced interaction with LC3 was sensitive to β-mercaptoethanol (Fig. 2j), indicating that disulfide bond formation underlies Nt-Arg-driven p62 interaction with LC3. These results collectively suggest that the N-end rule interaction of Nt-Arg with ZZ domain induces a conformational change, exposing PB1 domain and LC3 interaction region for polymerization and interaction with LC3, respectively.

### Development of small molecule ligands to p62 ZZ domain

**Fig. 3:**
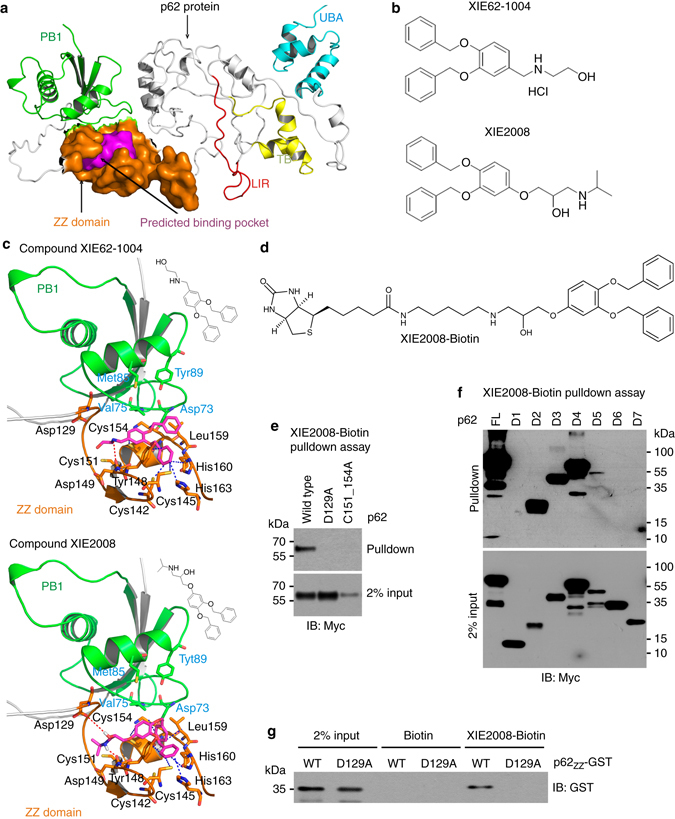
Development of small molecule ligands to p62 ZZ domain using 3D-modeling of p62 and virtual screening. **a** A 3D-model representing the structure of p62 that shows the predicted binding pocket present in ZZ domain. **b** The chemical structures of XIE62-1004 and XIE2008. **c** Docking model of p62 with XIE62-1004 and XIE2008. **d** The chemical structure of biotinylated XIE2008. **e** Pulldown assay using biotinylated XIE2008 and myc/His tagged ZZ point mutants expressed in HEK293 cells. 75 μg of total protein was used in pulldown assay, and p62 was detected by immunoblotting analysis using anti-Myc antibody. **f** Similar to **e** except that biotinylated XIE2008 pulldown assay was performed using myc/His tagged p62 deletion mutants. **g** Pulldown assay using biotinylated XIE2008 and 93-residue p62_ZZ_-GST containing intact ZZ domain

To develop biologically active ligands to p62, we employed 3D structure models of full-length p62 that were constructed and modeled using Modeller-9.12^47^ (Fig. 3a). Figure 3a showed that the predicted structure of ZZ domain revealed a putative pocket composed of two distinct compartments, hydrophobic (mainly formed by Cys142, Cys145, Tyr148, Leu159, His160, and His163) and hydrophilic (mainly formed by Asp129, Arg139, Asp147, and Asp149). Consistent with the computational results, the site-directed mutations of these residues impaired the ability of p62 to bind Nt-Arg. Virtual screening was carried out using an in-house chemical database of 540,000 compounds pre-filtered by our established cell-based partition chemistry-space matrix calculation algorithm^48^. Two small molecules, termed XIE62-1004 (docking score: 7.6) and XIE2008 (docking score: 7.7), showed high docking scores and predicted strong interactions with p62 ZZ domain (Fig. 3b, c). The docking results (Fig. 3c) illustrated that certain residues were more important for the recognition of p62 ligands, including Asp129, Cys142, Cys145, Tyr148, Asp149, Cys151, Cys154, His160, and His163. For example, Asp149 formed strong hydrogen bonds with both compounds (within 3.5 Å), while two other residues (Tyr148 and His160) formed π–π/hydrophobic interactions with both compounds. The only difference between XIE62-1004 and XIE2008 in the predicted binding patterns was that another hydrogen bond was found between Asp129 and XIE2008. Moreover, our model also predicted that some residues at PB1 domain also formed interactions with the ligands that bound to ZZ domain (Fig. 3c). Val75 and Met85 at PB1 domain formed hydrophobic interactions with these two compounds within 5 Å, while the distances between Asp73/Tyr89 at PB1 domain and our compounds were also within 5 Å. These results suggest that the binding of ZZ ligands (for example, X-peptide) would affect the spatial orientation of PB1 and its function. We synthesized XIE62-1004 (Supplementary Fig. 4a). Various docking parameters indicated that both compounds possessed certain functional groups structurally similar to type-2 ligands and shared structural interactions with the predicted pocket. To determine the direct binding of XIE2008 to p62 ZZ domain, we performed pulldown assays using biotinylated XIE2008 (Fig. 3d; Supplementary Fig. 4b) and full-length p62. The result showed that biotinylated XIE2008 bound wild-type p62 but not mutants carrying point mutations within ZZ domain, either D129 or C151/C154 (Fig. 3e). Moreover, pulldown assays using C-terminally (D1–D4) or N-terminally (D5–D7) deleted p62 mutants showed that biotinylated XIE2008 pulled down ZZ-containing fragments (D2, D3, D4, and D5) but not ZZ-lacking fragments (D1, D6 or D7) (Fig. 3f). Finally, we confirmed that biotinylated XIE2008 bound a 93-residue ZZ-only fragment (residues #83–175), p62_ZZ_-GST, but D129A-p62_ZZ_-GST that carries a point mutation within the ZZ domain (Fig. 3g).

### ZZ ligands induce p62 delivery to the autophagosome

**Fig. 4:**
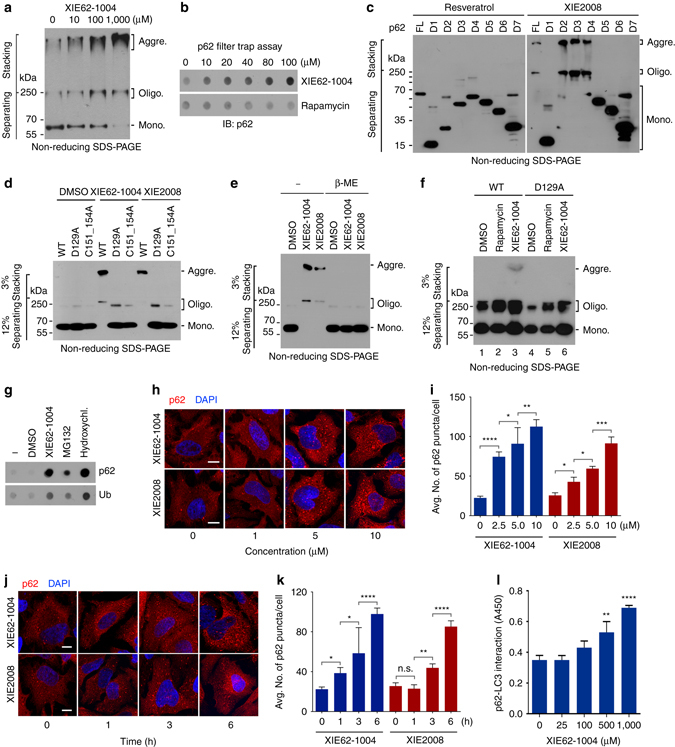
ZZ ligands induce self-polymerization and autophagy targeting of p62. **a** In vitro p62 oligomerization assay using HEK293 cell extracts expressing myc/His-tagged p62. After 2 h incubation of cell extracts with XIE62-1004 at room temperature, forms of p62 was detected by immunoblotting analysis using anti-Myc antibody following non-reducing SDS-PAGE. **b** In vitro filter trap assay of p62 using myc/His-tagged p62 expressed using the TnT lysate system. **c** In vitro oligomerization assay using myc/His tagged p62 deletion mutants, Forms of p62 were detected as described in **a**. **d** In vitro p62 oligomerization assay using myc/His-tagged p62 wild type and ZZ point mutants. **e** Similar to d except that in vitro p62 oligomerization assay was performed in the presence or absence of 50 mM β-mercaptoethanol. **f** In vivo p62 oligomerization assay using 1% Triton X-100 insoluble p62 (wild type and D129A mutant) expressed in HeLa cells treated with 5 μM XIE compounds for 24 h. **g** In vivo filter trap assay of 1% Triton X-100 insoluble p62. HeLa cells were treated with 10 μM XIE62-1004, 5 μM MG132 or 25 mM hydroxychloroquine for 16 h. **h** In vivo p62 puncta formation analysis employing immunocytochemistry. HeLa cells treated with XIE compounds for 12 h were stained for p62. Scale bar, 10 μm. **i** Quantification of **h**. Data are representative of three independent experiments, and values are expressed as the average number of p62 puncta per cell with the indicated S.D. Statistical significance was calculated using a one-way ANOVA test (*P < 0.05; **P < 0.01; ***P < 0.001; ****P < 0.0001). **j** Similar to **h**. Scale bar, 10 μm. **k** Quantification of **j**. Statistical significance was calculated using a one-way ANOVA test (n.s., *P* ≥ 0.05; *P < 0.05; **P < 0.01; ****P < 0.0001). **l** ELISA measuring the interaction of p62 with LC3 in the presence of XIE62-1004. Data represent the mean (±S.D.) of three independent experiments, each performed in triplicate. Statistical significance was calculated using a one-way ANOVA test (**P < 0.01; ****P < 0.0001)

To determine whether XIE62-1004 modulates p62 activities, we mixed HEK293 cell extracts with XIE62-1004 and monitored p62 aggregation in vitro. Non-reducing SDS-PAGE showed that XIE62-1004 induced the aggregation of p62 (Fig. 4a; Supplementary Fig. 5a). Filter-trap assay of p62 expressed in reticulocyte lysates also showed that XIE62-1004 induced the formation of p62 species which are trapped on the nitrocellulose membrane (Fig. 4b). A mapping analysis using serially deleted p62 fragments (D1 through D7) identified PB1 and ZZ domain to be required for p62 aggregation by XIE2008 (Fig. 4c) or XIE62-1004 (Supplementary Fig. 5b). Either of the point mutation D129A or C151/C154A in ZZ domain abolished the ability of p62 to undergo aggregation in response to the p62 ligands (Fig. 4d). As a control, we confirmed that p62 aggregation is not induced by known autophagic inducers such as rapamycin (Fig. 4b) and resveratrol (Fig. 4c). These results suggest that although Nt-Arg and other ZZ ligands induce the aggregation of p62 through their binding to ZZ domain, this process still requires PB1 domain. Finally, we found that high molecular weight p62 species were dissolved into monomers by β-mercaptoethanol (Fig. 4e), indicating that disulfide bond links p62 into high order aggregates. These results suggest that XIE62-1004 and XIE2008 act as synthetic N-degrons whose binding to p62 ZZ domain induces the aggregation of p62 in a manner similar to Nt-Arg of the N-end rule pathway.

To determine whether the p62 ligands modulate the activity of p62 in vivo, we treated HeLa cells expressing p62 with XIE62-1004 and performed non-reducing SDS-PAGE of cellular proteins which are insoluble in 1% Triton X-100 (Fig. 4f). The results showed that XIE62-1004 stimulated p62 aggregation in vivo (Fig. 4f, lanes 3 vs. 1). A point mutation, D129A, in ZZ domain abolished such an aggregation-inducing activity (Fig. 4f, lanes 6 vs. 3). Moreover, the autophagic blocker bafilomycin A1 increased the level of p62 aggregates in HeLa cells treated with XIE62-1004 (Supplementary Fig. 5c, lanes 6 vs. 3), indicating that the p62 aggregates are destined to lysosomal degradation. As an alternative approach, we treated HeLa cells with XIE62-1004 and performed the in vivo filter trap assay of cellular proteins which are insoluble in 1% Triton X-100, which showed the co-aggregation of p62 along with Ub-conjugated protein species (Fig. 4g). A similar aggregation pattern was observed with the proteasome inhibitor MG132 and the autophagic blocker hydroxychloroquine (HCQ) (Fig. 4g; Supplementary Fig. 5d). Blue native PAGE (BN-PAGE) showed that normally growing MEFs contain oligomeric p62 species (Supplementary Fig. 5e, f). Our results together suggest that XIE62-1004 and XIE2008 induce the formation of p62 aggregates in vivo which are destined to autophagic degradation.

It has been suggested that the oligomerization of p62 facilitates its delivery to the autophagosome^49^. We therefore examined whether our synthetic ZZ ligands facilitate the formation of p62 cytosolic puncta. Indeed, immunostaining of HeLa cells treated with XIE62-1004 or XIE2008 showed that both compounds efficiently induced the formation of cytosolic puncta positive for p62 at a concentration of 2.5 μM and in as early as 1 h (Fig. 4h–k). Ligand-induced formation of p62 foci was dose- and time-dependent (Fig. 4h–k). Next, we asked whether ZZ ligands modulate the interaction of p62 with LC3. Indeed, GST-pulldown assay coupled with ELISA showed that XIE62-1004 increased p62 interaction with LC3 (Fig. 4l). These results indicate that the binding of ligands to p62 ZZ domain commonly induces p62 self-oligomerization and aggregation and increases the interaction of p62 with LC3-II, most likely through an allosteric conformational change.

### p62 is an inducer of autophagy

**Fig. 5:**
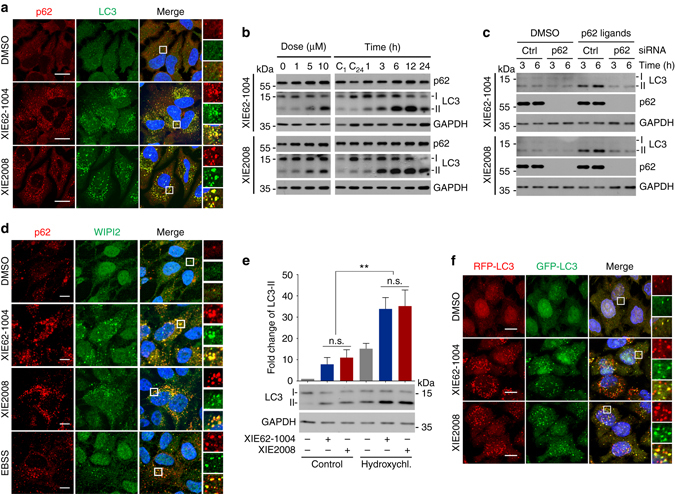
XIE62-1004 and XIE2008 induce autophagy. **a** In vivo autophagosome formation analysis in HeLa cells treated with 10 μM XIE62-1004 or XIE2008 for 16 h, followed by immunostaining of p62 and LC3. Scale bar, 20 μm. **b** Western blot analysis assessing LC3-I to LC3-II conversion induced by ZZ ligands. HeLa cells were treated with XIE62-1004 or XIE2008 for 12 h in a dose-dependent manner (*left*) or in a time-dependent manner at the concentration of 10 μM (*right*). C1 and C24, DMSO treatment for 1 h and 24 h, respectively. **c** Western blot analysis assessing p62-dependent conversion of LC3-I to LC3-II in ZZ ligand-stimulated HeLa cells. HeLa cells with or without p62 RNA interference were treated with 10 μM XIE62-1004 or 10 μM XIE2008. **d** In vivo WIPI puncta formation analysis in HeLa cells treated with 5 μM XIE62-1004 (12 h), 5 μM XIE2008 (12 h) or EBSS (2 h), followed by immunostaining of p62 and WIPI2. Scale bar, 10 μm. **e** Autophagic flux assay using HeLa cells pretreated with 10 μM XIE62-1004 or XIE2008 for 6 h followed by combination treatment with 25 μM hydroxychloroquine for 3 h. Data represent the mean (±S.D.) of three independent experiments. A one-way ANOVA was performed to assess statistical significance (n.s., *P* ≥ 0.05; **P < 0.01). **f** Autophagic flux assay using RFP–GFP–LC3 stably expressed in HeLa cells. The cells were treated with 10 μM XIE62-1004 and XIE2008 for 16 h

Selective autophagy mediates the degradation of cytotoxic materials, including misfolded protein aggregates, pathogens, and damaged mitochondria^50^. Once cells sense such cargoes, p62 collects and delivers the cargoes to autophagosomes. One outstanding question is how the activation of selective autophagy is synchronized with cargo formation. We therefore determined whether ligand-bound p62 induces selective autophagy. Indeed, immunostaining analysis showed that the induction of ligand-driven p62 puncta formation coincided with the stimulation of LC3-positive cytosolic puncta formation (Fig. 5a). Moreover, these two ligand-induced cytosolic puncta colocalized with each other, suggesting that ligand-bound p62 stimulated the formation of LC3-positive puncta (Fig. 5a). Consistently, immunoblotting analyses showed that XIE62-1004 and XIE2008 increased the synthesis of total LC3 and its conversion to a lipidated form, LC3-II (Fig. 5b). The ZZ ligands failed to induce the synthesis and activation of LC3 in p62 knockdown (Fig. 5c, lanes 6 vs. 8) as well as knockout cells (Supplementary Fig. 6a, lanes 6 vs. 8). These results suggest that the ZZ ligands induce autophagosome biogenesis.

It has been recently shown that WIPI2 plays an important role in the recruitment of the Atg12-Atg5-Atg16 complex to PtdIns(3)P-positive omegasomes, resulting in LC3-II formation^51^. To test whether our p62 ligands induces the formation of WIPI2-positive puncta, we performed immunostaining analysis of endogenous WIPI2 in HeLa cells treated with 10 μM XIE62-1004 or XIE2008 for 12 h. The results showed that the p62 ligands markedly increased the formation of WIPI2-positive puncta, which colocalized with p62-positive puncta (Fig. 5d). As a positive control, cells cultured in EBSS starvation media induced WIPI2 puncta in a fashion similar to the treatment of p62 ligands. These results suggest that the ligands to p62 ZZ domain induce the formation of WIPI2-positive omegasomes, leading to LC3-positive autophagosomes.

Cycloheximide degradation assay showed that autophagic induction was associated with accelerated degradation of p62 (Supplementary Fig. 6b). To confirm that the increased formation of LC3 puncta is not due to reduced autophagic flux, we treated HeLa cells with various blockers of autophagic flux, such as NH_4_Cl, bafilomycin A1, and HCQ. When co-treated with XIE62-1004, autophagic blockers synergistically increased the synthesis and activation of LC3 (Fig. 5e; Supplementary Fig. 6c, lanes 3 vs. 4), indicative of stimulated autophagic flux. As an alternative way to measure autophagic flux, we monitored the ratio of RFP vs. GFP fluorescent signals in HeLa cells stably expressing RFP–GFP–LC3. In this assay system based on differential pH stability of RFP (acid resistant) and GFP (acid sensitive), RFP-GFP-LC3 generates yellow fluorescent signals in autophagosomes (neutral pH) and red fluorescent signals in autolysosomes (acidic pH). Fluorescence analysis revealed co-distribution of LC3 in autophagosomes (*yellow*; RFP^+^GFP^+^) and autolysosomes (*red*; RFP^+^GFP^−^) (Fig. 5f; Supplementary Fig. 6d). These results suggest that upon binding to a ZZ ligand (e.g., Nt-Arg of arginylated proteins), p62 acts as an inducer of selective autophagy, providing a novel mechanism that links cargo formation to autophagic core machinery.

### ZZ ligands facilitates degradation of Huntingtin aggregates

**Fig. 6:**
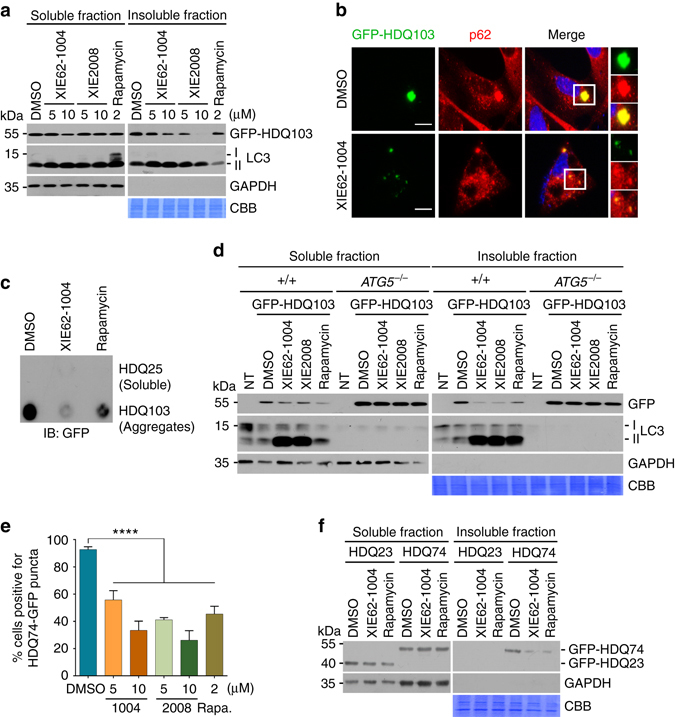
XIE62-1004 and XIE2008 accelerate autophagic clearance of mutant huntingtin protein aggregates (mHTT). **a** Stimulated degradation of GFP-HDQ103 induced by XIE compounds. HeLa cells transiently expressing GFP-HDQ103 were treated with XIE62-1004 (1004), XIE2008 or rapamycin for 18 h and fractionated into soluble and insoluble proteins in 1% Triton X100, followed by immunoblotting analysis. **b** Inhibition of inclusion body formation by XIE62-1004. HeLa cells expressing GFP-HDQ103 were treated with 10 μM XIE62-1004 for 18 h and analyzed by immunofluorescent analysis of GFP-HDQ103 and immunostaining of p62. **c** Inhibition of HDQ103 aggregate formation by XIE62-1004. HeLa cells transiently expressing GFP-HDQ25 or GFP-HDQ103 were treated with 10 μM XIE62-1004 or 2 μM rapamycin for 18 h, followed by filter trap analysis. **d** Facilitated autophagic clearance of HDQ103 aggregates by XIE compounds. Wild-type and *ATG5* ^*−/−*^ MEFs transiently expressing GFP-HDQ103 were treated with 10 μM XIE62-1004 (1004), 10 μM XIE2008 (2008) or 2 μM rapamycin for 18 h, followed by soluble and insoluble fractionation and immunoblotting analyses. **e** Inhibition of HDQ74-GFP inclusion body formation by XIE compounds. Inducible PC12 cells stably expressing EGFP-HDQ74 (mutant Htt) were treated with 1 μg/ml doxycycline for 8 h followed by stimulation with XIE62-1004 (1004), XIE2008, or rapamycin for 18 h and subjected to fluorescence analysis of GFP. Average percentage of cells positive for HDQ74-GFP puncta was calculated by counting 100 cells per experimental condition in each experiment. Data represent the mean (±S.D.) of three independent experiments. Statistical significance was calculated using a one-way ANOVA test (****P ≤ 0.0001). **f** Enhanced autophagic degradation of GFP-HDQ74 in XIE62-1004 stimulated PC12 cells. Inducible PC12 cells stably expressing EGFP-HDQ23 or EGFP-HDQ74 were treated with 10 μM XIE62-1004 or 2 μM rapamycin for 18 h following induction with 1 μg/ml doxycycline for 8 h and fractionated into soluble and insoluble proteins

Huntington’s disease is a neurodegenerative disorder caused by the aggregation of mutant huntingtin (mHTT) proteins containing glutamine repeats, called polyQ tract^52^. The aggregated form of mHTT is at least in part resistant to all known degradative systems, including the UPS and macroautophagy^9^. We therefore determined whether pharmacological activation of macroautophagy using ZZ ligands facilitates the degradation of mHTT. HeLa cells transiently expressing GFP-HDQ103, a mHTT carrying 103 glutamine repeats, were treated with XIE62-1004 or XIE2008 in comparison with rapamycin, an inhibitor of mammalian target of rapamycin (mTOR). Cellular proteins were separated into soluble and insoluble fractions in 1% Triton X-100. A significant portion of GFP-HDQ103 was retrieved in the insoluble fraction (Supplementary Fig. 7b, c, lane 4). Immunoblotting and dot blot analyses showed that XIE62-1004 and XIE2008 reduced the levels of GFP-HDQ103 aggregates in the insoluble fraction (Fig. 6a, c). In immunostaining analysis, GFP-HDQ103 was deposited to the aggresome near the nucleus, which colocalized with p62 (Fig. 6b; Supplementary Fig. 7a). The treatment of p62 ligands resulted in a marked reduction in GFP-HDQ103 inclusions as well as associated p62 signals (Fig. 6b; Supplementary Fig. 7d). XIE62-1004 and XIE2008 failed to induce the degradation of GFP-HDQ103 in *ATG5* ^*-/-*^ MEFs (Fig. 6d), suggesting that these ligands exert their efficacy through autophagic induction. Similar effects were obtained with cells stably expressing mutant HDQ74 aggregates (Fig. 6e, f). These results suggest that p62 ligands accelerate autophagic degradation of mHTT.

### p62 mediates crosstalk between the UPS and autophagy

The UPS and autophagy have long been thought to be independent of each other. This paradigm was challenged by the findings that cells activate autophagy when the UPS is impaired^13, 53^. Extensive studies subsequently showed that protein quality control under various stresses needs timely cooperation between the UPS and autophagy in a real-time basis^2, 10–12^. To date, the key modulators underlying the crosstalk between the UPS and autophagy remain to be identified.

**Fig. 7:**
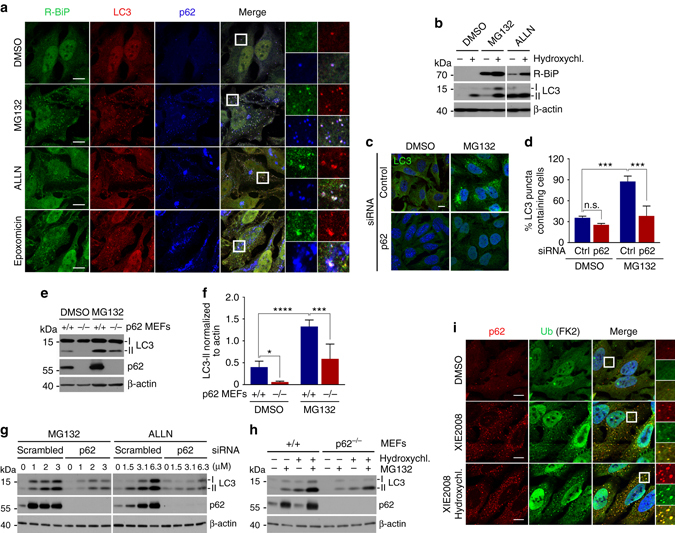
p62 mediates crosstalk between the UPS and autophagy through the N-end rule interaction with Nt-Arg. **a** Delivery of p62-associated R-BiP to autophagosomes in proteasome inhibited cells. HeLa cells were treated with 10 μM MG132, 25 μM ALLN or 1 μM epoxomicin for 18 h, followed by immunostaining analysis of R-BiP, LC3, and p62. **b** Autophagic flux analysis of R-BiP in HeLa cells treated with DMSO or MG132 in the absence or presence of 25 μM hydroxychloroquine for 24 h. **c** Autophagosome formation analysis. HeLa cells transfected with either control or p62 siRNA for 40 h were treated with 5 μM MG132 for 8 h and subjected to immunostaining of LC3. **d** Quantification of **c**. The graph represents the average percentage (±S.D.) of cells containing more than 15 LC3 positive puncta (*n* = 3). Average of 100 cells was counted per each experimental condition in each experiment. Data represent the mean (±S.D.) of three independent experiments. Statistical significance was calculated using a two-way ANOVA test (n.s., *P* > 0.05; ***P < 0.001). **e** Inhibition of MG132 induced autophagosome formation by p62 deficiency. Wild-type and *p62* ^*−/−*^ MEFs were treated with 1 μM MG132 for 9 h, followed by immunoblotting analysis. **f** Quantification of **e**. The intensity of LC3-II band was normalized to β-actin. Data represent the mean (±S.D.) of three independent experiments. Statistical significance was calculated using a two-way ANOVA test (*P ≤ 0.05; ***P < 0.001; ****P < 0.0001). **g** HEK293 cells were transfected with control or p62 siRNA and incubated with proteasome inhibitors, MG132 and ALLN, at concentrations indicated in the figure for 16 h, followed by immunoblotting. **h** Analysis of autophagosome biogenesis using p62 wild type and knockout MEFs that were treated with 1 μM MG132 alone or combination with hydroxychloroquine for 9 h. **i** Stimulated autophagic clearance of p62 cargoes in XIE2008 stimulated cells. HeLa cells were untreated or treated with 5 μM XIE2008 alone or in combination with 25 μM hydroxychloroquine for 8 h, followed by immunostaining analysis with anti-p62 and Ub conjugates-specific FK2 antibodies

To determine whether p62 is such a modulator in the crossroad between the UPS and autophagy, we treated cells with the proteasomal inhibitor MG132 and monitored autophagic activities and the formation of R-BiP, a physiological substrate and ligand of p62^11^. We have recently shown that R-BiP is the Nt-arginylated form of BiP, an ER-residing chaperone, and binds p62^11^. The results showed that proteasomal inhibition induced both autophagy and R-BiP (Fig. 7a, b; see Fig. 7b, lanes 3 vs. 4, lanes 5 vs. 6). Consistent with our earlier finding^11^, R-BiP formed cytosolic puncta that colocalized with p62 puncta as well as LC3 puncta in response to proteasome inhibition (Fig. 7a), suggesting that R-BiP, induced by reduced UPS activities, is associated with p62 and delivered to the autophagosome. Autophagy flux assays showed that these R-BiP species are at least in part degraded by autophagy (Fig. 7b; lanes 3 vs. 4 and 5 vs. 6; Supplementary Fig. 8a, b). Loss of p62 resulted in the metabolic stabilization of the recombinant R-BiP protein, R-BiP^Δ^-GST, which is generated when Ub-R-BiP^19-124^-GST (Ub-R-BiP^Δ^-GST) is cotranslationally cleaved by deubiquitinating enzymes (Supplementary Fig. 8c; lanes 5 vs. 2). Moreover, the mutation of Nt-Arg to valine (Val) protected BiP from degradation as determined by anti-GST immunoblotting (Supplementary Fig. 8c; lanes 3 vs. 2). These results together suggest that p62 is an N-recognin, the first to be identified, of the autophagic N-end rule pathway.

Next, we asked whether p62 is essential to activate autophagy under proteasomal inhibition. Indeed, HeLa cells lacking p62 failed to properly induce autophagosome formation in response to proteasomal inhibition with MG132 (Fig. 7c). The percentage of LC3 puncta containing cells were greatly diminished in MG132-stimulated HeLa cells deficient in p62 (Fig. 7d). Cells deficient in p62 also showed a significant reduction of MG132-induced autophagosome formation, as judged by the reduced level of LC3-II (Fig. 7e, f, lanes 3 vs. 4, Fig. 7d; Supplementary Fig. 8d, e). Knocking down p62 in HEK293 cells inhibited the optimal induction of autophagosome formation by not only MG132 but also other various proteasomal inhibitors such as ALLN, bortezomib, and epoxomicin (Fig. 7g; Supplementary Fig. 8f–h), further supporting the role of p62 in proteasomal inhibition-induced autophagosome formation. Blocking autophagy flux by HCQ confirmed that p62 is required for proteasomal inhibition-induced LC3 expression and autophagosome biogenesis (Fig. 7h; Supplementary Fig. 8i). Consistently, p62 ligands accelerated the formation of Ub-conjugates in p62-positive puncta (Fig. 7i), indicating that p62 activation stimulated autophagic delivery of Ub-conjugates to autophagosomes. These results suggest that p62 mediates crosstalk between the UPS and autophagy through binding to N-degrons, such as Nt-Arg.

## Discussion

**Fig. 8:**
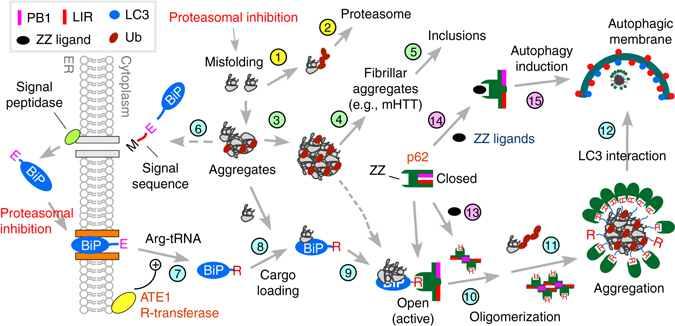
A model illustrating the N-end rule pathway in the modulation of autophagy. Cytosolic misfolded proteins are normally degraded through ubiquitination and proteasomal degradation (Steps 1 and 2). Some misfolded proteins (e.g., mHTT) may be prone to aggregation (Step 3) and, thus, are difficult to be degraded by the proteasome. In neurodegenerative diseases, these proteasome-resistant oligomeric aggregates grow into fibrillar forms and large inclusions (Steps 4 and 5). In protein quality control, cells sense the accumulation of such misfolded proteins and their aggregates, inducing Nt-arginylation of ER-residing proteins, such as BiP (Steps 6 and 7). Nt-arginylated ER proteins accumulate in the cytosol. Cytosolic R-BiP is associated with misfolded protein cargoes (Step 8) and binds the ZZ domain of p62 through its Nt-Arg (Step 9). Upon binding to Nt-Arg, p62 undergoes a conformational change, exposing PB1 and LC3-interaction domain (Step 10). This accelerates self-polymerization of p62, resulting in the formation of cargo–R-BiP–p62 protein aggregates (Step 11). The binding with Nt-Arg also enhances p62 interaction with LC3 on autophagic membranes (Step 11), leading to the delivery of cargo–R-BiP–p62 complexes into autophagosomes (Step 12). In this study, we developed small molecule ligands to p62 ZZ domain. Our results suggest that ligand-bound p62 acts as an autophagic inducer (Steps 14 and 15) by enhancing the synthesis of LC3-I and its conversion into LC3-II and promoting the formation of autophagosomes

In this study, we show that the N-end rule pathway mediates autophagic proteolysis of misfolded proteins. In the autophagic N-end rule pathway, whose model is proposed in Fig. 8, p62 is an N-recognin that binds to various type-1 and type-2 residues, including Nt-Arg. Upon binding to Nt-Arg, p62 undergoes a conformational change, which exposes PB1 and LC3-interaction domain (Step 9). This accelerates the self-polymerization of p62 (Step 10) and enhances the interaction of p62 with LC3 on autophagic membranes (Step 11), leading to the delivery of cargo–p62 complexes into autophagosomes (Step 12). It is notable that the binding affinity (*K*_D_, 44 nM) of p62 to a single N-terminal residue, Nt-Arg, is 50–80 fold higher than that of UBR1 (*K*_D_, 1.6–3.2 μM)^19^, suggesting that Nt-arginylated proteins forms a stable complex with p62 throughout autophagic delivery and degradation. Thus, in the autophagic N-end rule pathway, Nt-Arg functions as a delivery and degradation determinant as well as an activating ligand to p62. It should be determined to what degree p62-dependent proteolysis and other autophagic processes are mediated through this biochemical mechanism.

Selective autophagy is normally maintained at the basal level and induced when cytotoxic materials are generated or invade into the cell. The pathways that link cytotoxic materials to autophagic induction are the subject of extensive studies. Our earlier^14^ and current studies show that Nt-arginylation of ER-residing proteins (possibly many cytosolic proteins as well) is selectively induced by various stresses (Step 6), such as proteasomal inhibition, which was invariably associated with autophagic induction. Thus, one of the physiological signals that induce Nt-arginylation is the formation of misfolded proteins redirected from the UPS to autophagy (Steps 1–5). Given our finding (Fig. 2) that Nt-Arg acts as an activating ligand to p62 (Fig. 8, Step 9), we propose that the increased cellular concentration of Nt-Arg (of arginylated substrates) is a stress signal that activates p62 in response to cytotoxic materials. Our results also suggest that p62 may normally be present as an inactive, closed form to minimize off-target degradation of innocent molecules and, only when needed, structurally activated via Nt-arginylation (Steps 9 and 10), leading to the oligomerization of p62. Consistent with our results, previous studies have shown that oxidative stress correlates to the formation of covalently crosslinked p62 oligomers^54, 55^. Recent studies have shown that the phosphorylation of p62 is involved in the regulation of protein quality control by autophagy^56, 57^. The functional relationship of the Nt-Arg–p62 interaction with other known regulatory mechanisms of p62 such as the phosphorylation needs to be further investigated.

One fundamental question in autophagy is how cells induce autophagosome formation in response to cytotoxic materials. For example, when p62 collects misfolded protein aggregates, autophagosomes should have been ready to accept cargo–p62 complexes. How do the cells synchronize the two events, cargo recognition/collection/condensation and autophagosome biogenesis? In this study, we developed small molecule ligands to the ZZ domain of p62 (Fig. 3). We made an unexpected finding that ligand-bound p62 acts as an autophagic inducer (Fig. 8, Steps 14 and 15), enhancing the synthesis of LC3 and its conversion into LC3-II and promoting autophagosome formation. Despite the pivotal role of p62 in macroautophagy, the active role of p62 in the modulation of autophagic core machinery, such as ATG proteins and autophagosomes, has not been appreciated. Our results indicate that p62 is a critical regulator of autophagy, which is essential to induce autophagosome biogenesis in response to the formation of its cargoes.

Another important subject in the field of protein degradation is the mechanism underlying the crosstalk between the UPS and autophagy. This crosstalk is considered to be a compensatory mechanism to maintain proteostasis during stress responses. Although a large number of studies proposed molecules or pathways, including the unfolded protein response (UPR)^58^, p53^59^, AMPK^60^, and HDAC6^53^, unfortunately, there is no consensus yet. Our results collectively identify p62 to be such an important regulator in the crosstalk. As illustrated in Fig. 8, we propose a circuit in which the impairment of the UPS induces Nt-arginylation (Step 6), resulting in the increased cellular concentration of Nt-Arg (Step 7). The resulting Nt-Arg residue of many arginylated proteins, including R-BiP, bind to the ZZ domain of p62 (Step 9), enabling cargo collection (Step 8) through a conformational change (Step 9). Nt-Arg-bound p62, in turn, acts as an autophagy inducer (Step 15). Through this circuit (Steps 6–15), autophagy can be induced in response to reduced UPS activities.

Autophagic induction in pathogenic conditions could have substantial benefits of therapy, including the degradation of cytotoxic materials, such as misfolded protein aggregates. Many small molecules that induce autophagy are now available. Rapamycin is a representative autophagy inducer that exerts its efficacy by inhibiting mTOR^61, 62^. However, because of the diverse biological activities of mTOR, rapamycin has limitation in therapeutic application. In this study, we successfully used p62 ligands to accelerate the autophagic degradation of mHTT protein aggregates in cultured cells (Fig. 6). While the results should be validated in vivo, our results indicate that p62-mediated induction of autophagy may have therapeutic application in various biological processes where autophagic induction is beneficial.

## Methods

### Antibodies for immunoblotting and immunostaining

The antibodies used in this study are as follows: mouse monoclonal anti-p62 (Abcam, ab56416, 1: 600,000), mouse monoclonal anti-GST (Santa Cruz, sc-138, 1:2000), mouse monoclonal anti-Myc (9E10, in house, 1:5000), mouse monoclonal anti-actin (Sigma, A5316, 1:15,000), mouse monoclonal anti-FK2 specific to Ub-conjugated proteins (Millipore, 04-263, 1:1000), mouse monoclonal anti-ATE1 (Santa Cruz, sc-271219, 1:1000), rabbit polyclonal anti-LC3 (Novus Biologicals, NB100-2220, 1:1000), rabbit polyclonal anti-LC3 (Sigma, L7543, 1:1000), rabbit polyclonal anti-R-BiP (AbFrontier, 1:1000), rabbit polyclonal anti-His (MBL International, pm032, 1:1000), rabbit polyclonal anti-GAPDH (Santa Cruz, sc-25778, 1:2000), goat polyclonal anti-UBR2 (Novus Biologicals, 1:1000, NBP1-45243), mouse monoclonal anti-GFP (Santa Cruz, sc-9996, 1:2000), and mouse monoclonal anti-WIPI2 (Abcam, ab105459, 1:5000). The following secondary antibodies were used: alexa fluor 488 goat anti-rabbit IgG (Invitrogen, A11029, 1:200), texas red goat anti-mouse IgG (Invitrogen, T6390, 1:500), anti-rabbit IgG-HRP (Cell Signaling, 7074, 1:10,000), and anti-mouse IgG-HRP (Cell Signaling, 7076, 1:10,000).

### Plasmids and other reagents

The p62-expressing construct was generated by PCR amplification of a human *p62* cDNA fragment from the hMU012675 clone (21C Frontier Human Gene Bank) and subcloned into the pcDNA 3.1/*myc*-His plasmid (Invitrogen) at Eco*R*I/XhoI sites. This method was also used to generate a series of p62 domain deletion mutants designated D1–D8, CD1–CD9, and ND1–ND6. Site-directed mutagenesis was employed to generate amino acid substitutions (X to Ala) in the p62 ZZ domain. Six ZZ mutants were subcloned as described above. These plasmids were transiently transfected using Lipofectamine 2000 (Invitrogen). Other reagents used in this study were ammonium chloride, bafilomycin A1, and HCQ (Sigma); glutathione coated plates, TBM substrate and high capacity streptavidin agarose resin (Thermo Fisher Scientific); RNA*i*Max (Invitrogen); GST-tagged LC3 recombinant protein (Enzo Lifescience); and TNT coupled reticulocyte system (Promega).

### Cell culture and immunoblotting

HEK293 and HeLa cells as well as +/+ and *p62* ^*−/−*^ MEFs were cultured in DMEM (GIBCO) supplemented with 10% FBS (HyClone) in a 5% CO_2_ incubator. HEK293 and HeLa cells were directly purchased from ATCC. +/+ and *ATG5* ^*−/−*^ MEFs were obtained from RIKEN (Japan). +/+ and *p62* ^*−/−*^ MEFs were obtained from Keiji Tanaka’s laboratory (Tokyo Metropolitan Research Institute, Japan) with Tetsuro Ishii’s permission. p62 and ATG5 knockout MEFs were verified using immunoblotting analysis. We regularly test cell lines for mycoplasma contamination. For immunoblotting, cells were lysed in buffer A (20 mM HEPES, pH 7.4, 0.5% Triton X-100, 0.5% CHAPS, and 10% glycerol) or alternatively buffer B (0.05 M Tris-HCl. pH 8.0, 0.15 M NaCl, 5.0 mM EDTA, and 1% NP-40) with protease and phosphatase inhibitor cocktail tablets (Roche). Whole cell lysates were separated by SDS-PAGE and transferred onto polyvinyllidene difluoride membranes.

### X-peptide pulldown assay

Plasmids expressing p62 mutant constructs were transiently transfected into HEK293 cells using Lipofectamine 2000. After 24 h, trypsinized cells were collected in growth medium and centrifuged. The cell pellets were resuspended in a hypotonic buffer (10 mM HCl, 1.5 mM MgCl_2_, and 10 mM HEPES, pH 7.9) and incubated in ice for 30 min. The cell suspensions were subjected to five freeze-thaw cycles or dounce homogenization, followed by centrifugation at 15,928 × *g* at 4 °C for 10 min. In the X-peptide pulldown assay, a set of 12-mer peptides (X-I-F-S-T-I-E-G-R-T-Y-K-biotin) bearing N-terminal type 1 (Arg, His, and Lys), type 2 (Phe, Trp, Tyr, and Leu) or stabilizing (Val, Asp, and Gly) residues were cross-linked through C-terminal biotin to streptavidin agarose resin (0.5 mg peptide per ml settled resin). The X-peptide beads were diluted in five volumes of PBS and incubated overnight at 4 °C. The beads were centrifuged at 1000 × *g* for 3 min and washed three times in an equal volume of PBS. Soluble HEK293 extracts (30 µl) containing 150–200 µg of total protein were diluted in 300 µl binding buffer (0.05% Tween 20, 10% glycerol, 0.2 M KCl, and 20 mM HEPES, pH 7.9) and mixed with X-peptide beads (50 µl packed volume). The mixtures were incubated at 4 °C for 2 h with gentle rotation. The beads were pelleted by centrifugation at 1000 × *g* for 30 s, washed five times with 1 ml of binding buffer at 4 °C for 20 min, resuspended in 20 µl SDS sample buffer, and heated at 100 °C for 5 min. Analysis was performed by SDS-PAGE and immunoblotting.

### Surface plasmon resonance assay

The SA sensor chip (Biacore) with pre-immobilized streptavidin was saturated with biotinylated-X-peptides (X=Arg, Val or Phe) by injecting 140 μl of biotinylated X-peptides (10 µM) into a flow cell for 10 min at a 10 μl/min rate. To analyze the binding kinetics, p62-D3-GST inserted in pGEX4T1 was expressed and purified in *Escherichia coli*. Various concentrations of p62-D3-GST were diluted in HBS-EP buffer (0.01 M HEPES, pH 7.4, 0.15 M NaCl, 3 mM EDTA, and 0.005% Surfactant P20) and injected onto the sensor chip for 150 s at 30 µl/min, and the response unit (RU) was recorded. After injecting the analyte, HBS-EP buffer was poured over the chip for 420 s at 30 µl/min to allow the bound analytes to dissociate from the immobilized X-peptide and dissociation curves were obtained. The RU elicited by injecting 1% elution buffer (20 mM HEPES, pH 7.0, 0.2 M NaCl, 10% glycerol, and 2 mM DTT) included HBS-EP buffer and was used as the vehicle control. Biacore X-100 control software was used to measure the changes in RU and to plot the binding curve. The curves obtained from the surface plasmon resonance assay experiments were analyzed and the dissociation equilibrium constant (*K*_D_) of p62-D3-GST to immobilized biotinylated peptides was calculated and derived from the equation, *K*_D_ = *k*_d_/*k*_a_, where *k*_d_ and *k*_a_ are dissociation- and association-rate constants, respectively.

### Molecular modeling of p62

The 3D structure model of full-length human p62 protein was constructed using Modeller-9.12^47^ with multiple sequence alignments and homology modeling protocol^63, 64^. The models were further refined by clustering, docking and molecular dynamics (MD) simulation. For example, we first performed 50 ns MD for conformational sampling. We also carried out a series of p62 dockings with the reported interacting partners, including IDR-1, LC3, Keap1, λPKC, and ubiquitin. As a result, the best 3D structure of p62 protein was then used in virtual screening and molecular docking studies.

### Virtual screening for chemical ligands to p62 ZZ domain

The putative binding cavity of p62 ZZ-domain was predicted using Fast Connolly Type implemented in the MOLCAD module in SYBYL-X 1.3 by a similar protocol as described previously^65^, before which the corresponding ZZ domain residues were first selected to generate a protocol with the default threshold radius of 0.5. Several focal residues involved in our predicted binding pocket included Asp129, Asn132, Val135, Arg139, Cys142, Cys145, Asp147, Tyr148, Asp149, Cys151, Cys154, Lys157, Leu159, His160, and His163. Docking virtual screening searches were carried out to screen a pre-filtered in-house database of 540,000 compounds with our published cell-based partition chemistry-space matrix calculation algorithm^48, 66–68^, in which the docking score was expressed in -log_10_(*K*_d_). The top-ranked compounds with the docking scores greater than 7.0 were selected for further experimental validation studies in vitro. Among all compounds tested, two small molecules (XIE62-1004 and XIE2008), which showed very high docking scores, were the most promising compounds.

### Chemical synthesis and analytical data

Synthesis and analytical data of 3,4-Bis(benzyloxy)benzaldehyde **2**. To the stirred solution of 3,4-dihydroxybenzaldehyde (3.00 g, 21.7 mmol) in dry DMF (50 mL) added anhydrous K_2_CO_3_ (9.60 g, 69.4 mmol) followed by benzyl bromide (7.65 g, 44.7 mmol). The resulted mixture was stirred at room temperature for 2 h. Additional K_2_CO_3_ (2.4 g, 17.3 mmol) was added, and the mixture was heated to 70 °C for 30 min and then cooled to room temperature. The mixture was partitioned between H_2_O and ether (120 ml each). The organic layer was separated, and the water layer was extracted with ether (3 × 50 ml). The pooled organic layers were washed with H_2_O (2 × 50 ml) and saturated aqueous NaCl (50 mL). The pale, straw-colored extracts were dried over anhydrous sodium sulfate and concentrated to yield 3,4-Bis(benzyloxy)benzaldehyde **2** (6.57 g, 95%) as a cream-colored solid after washing with hexanes (75 ml) and drying. ^1^H NMR (400 MHz, CDCl_3_): *δ* 9.81 (s, 1H), 7.49–7.31 (m, 12H), 7.04 (d, *J* = 8.3 Hz, 1H), 5.27 (s, 2H), 5.22 (s, 2H).

Synthesis and analytical data of 2-((3,4-bis(benzyloxy)benzyl)amino)ethan-1-ol **3**: 3,4-Bis(benzyloxy)benzaldehyde **2** (3.18 g, 10 mmol) was dissolved in dry ethanol (20 ml), and ethanolamine (0.61 g, 10 mmol) was added. The reaction mixture was stirred for 12 h at 60 ^o^C. The reaction solution was cooled to room temperature. NaBH_4_ (0.57 g, 15 mmol) was added slowly in portions, and the resulting solution was stirred for another 12 h. The solvent was evaporated in vacuum, and the residue was dissolved in water and extracted with ethyl acetate. The organic layers were combined and dried with anhydrous Na_2_SO_4_, filtered, and evaporated in vacuum. The residue was purified by flash column to generate the desired product 2-((3,4-bis(benzyloxy)benzyl)amino)ethan-1-ol **3** (2.0 g, 56%). ^1^H NMR (400 MHz, CDCl_3_): *δ* 7.52–7.33 (m, 10H), 7.01–6.84 (m, 3H), 5.20 (s, 2H), 5.17 (s, 2H), 3.71 (s, 2H), 3.64 (t, *J* = 4.8, 2H), 2.93 (s, 2H), 2.72 (t, *J* = 4.8, 2H).

Synthesis and analytical data of XIE62-1004: 2-((3,4-bis(benzyloxy)benzyl)amino)ethan-1-ol **3** (1.0 g, 2.75 mmol) was dissolved in absolute methanol (25 mL), and pumped HCl gas for 1 h. The mixture was stirred for another 2 h, evaporated to about 1 mL and added hexane to get the solid, which was filtered and dried to give the final compound XIE62-1004 (720 mg, 65%). ^1^H NMR (400 MHz, DMSO-d6): *δ* 8.83 (bs, 2H), 7.52–7.46 (m, 5H), 7.31–7.32 (m, 1H), 7.26–7.20 (m, 5H), 7.12–7.10 (m, 1H), 7.05–7.03 (m, 1H), 5.24–5.22 (m, 1H), 5.14 (s, 2H), 5.11 (s, 2H), 4.07 (s, 2H), 3.67–3.63 (m, 2H), 2.90 (s, 2H). ^13^C NMR (400 MHz, CDCl_3_): *δ* 149.0, 148.2, 137.4, 133.3, 128.5, 127.8, 127.5, 127.4, 121.3, 115.4, 115.2, 71.5, 71.3, 60.8, 53.2, 50.7. LC−MS (ESI): *m*/*z* 364.3 (M + H)^+^.

### Synthesis and analytical data of XIE2008-Biotin

m-Chloroperbenzoic acid (0.81 g, 4.71 mmol) was added to a solution of the above synthesized 3,4-bis(benzyloxy)benzaldehyde **2** (1.0 g, 3.14 mmol) in dichloromethane (15 mL). The mixture was stirred at room temperature for 24 h and then diluted with ethyl acetate (50 mL). The organic solution was successively washed with saturated aqueous Na_2_CO_3_ solution and brine. The solvent was evaporated in vacuo to give 3,4-bis(benzyloxy)phenyl formate. NaOH (6 N) was added to a stirred solution of crude 3,4-bis(benzyloxy)phenyl formate in MeOH (15 mL). After stirring at room temperature for 10 min, 10% aq. HCl solution was added. The reaction mixture was diluted with ethyl acetate (50 mL), washed with brine and dried over anhydrous Na_2_SO_4_. The flash chromatography (7:3 hexane/ethyl acetate) furnished the compound 3,4-bis(benzyloxy)phenol **3** as solid (0.77 g, 80 % yield). A solution of 3,4-bis(benzyloxy)phenol **3** (0.5 g, 1.63 mmol) in EtOH (6 mL) was treated with potassium hydroxide (91 mg, 1.63 mmol) and epichlorohydrin (151 mg, 1.63 mmol) and continued stirring at ambient room temperature for 6 h and then concentrated in vacuum. The residue was suspended in water, and the mixture extracted with ether and dried with anhydrous Na_2_SO_4_ and evaporated in vacuum to give 2-((3,4-bis(benzyloxy)phenoxy)methyl)oxirane **4** (414 mg, 70%). A solution of 2-((3,4-bis(benzyloxy)phenoxy)methyl)oxirane **4** (15 mg, 0.004 mmol) in MeOH (2 mL) with EZ-Link Pentylamine-Biotin (13 mg, 0.004 mmol) was refluxed for 6 h. After rotary evaporation of the mixture, to remove traces of EZ-Link Pentylamine-Biotin azeotrope with toluene (2 × 5 mL) and the residue was dissolved in methanol and added oxalic acid dehydrate. After evaporation, the residue was recrystallized from methanol/ether to give XIE2008-Biotin (17 mg, 60%) as a white solid and the final purity of the XIE2008-Biotin (>98%) was assessed by RP-HPLC on an analytical Vydac C_18_ column (4.6 mm × 250 mm, 300 Å, 5 μm particle size). The molecular masses of purified XIE2008-Biotin confirmed by recording its mass spectrum, by its molecular ion peak at *m*/*z* 691.74 [M + H]^+^ corresponds to molecular formula C_38_H_50_N_4_O_6_S.

### Soluble and insoluble fractionation

HeLa cells were transiently transfected with plasmids encoding either wild-type (Q25) or mutant (Q103) huntingtin gene using X-tremeGENE HP. Following transfection, cells were cultured for 8 h and treated with DMSO, XIE62-1004, XIE2008 or rapamycin for 16 h. The cells were pelleted, resuspended in a cell lysis buffer (20 mM HEPES pH 7.9, 0.2 M KCl, 1 mM MgCl_2_, 1 mM EGTA, 1% Triton X-100, 10% glycerol, protease inhibitor and phosphatase inhibitor) and incubated on ice for 30 min. Soluble- and insoluble-fractions in 1% Triton X-100 were obtained by centrifugation at 13,000 × *g* for 20 min at 4 °C. The insoluble fraction was solubilized in a SDS-detergent lysis buffer (20 mM HEPES pH 7.9, 0.2 M KCl, 1 mM MgCl_2_, 1 mM EGTA 1% Triton X-100, 1% SDS, 10% glycerol, protease inhibitors, and phosphatase inhibitors). After sonication, the samples were heated at 100 °C for 10 min in SDS sample buffer and subjected to western blotting analysis.

### Immunocytochemistry

HeLa cells were cultured on cover slips and fixed with 4% paraformaldehyde in PBS (pH 7.4) for 10 min at room temperature. After washing three times with PBS, the cells were permeabilized with ice-cold methanol for 2.5 min. After three washes with PBS, the cells were incubated with blocking solution (5% BSA in PBS) for 1 h and then with primary antibody overnight at 4 °C. The next day, the cells were washed five times for 5 min each time with PBS and then incubated with secondary antibody for 1–2 h. The cells were washed four times with PBS for 5 min each time, and DAPI stained for 15 min. After three washes with PBS, the coverslips were mounted on slides using Fluoro-GEL (Electron Microscopy Sciences). Confocal images were taken by laser scanning confocal microscope 510 Meta (Zeiss) and analyzed using Zeiss LSM Image Browser (ver. 4.2.0.121). To investigate the effect of XIE62-1004 and XIE2008 compounds on p62 puncta formation, cells were incubated with different concentrations of XIE compounds (0, 1, 2.5, 5, and 10 μM) for 12 h or incubated with 10 µM XIE compound for varying time periods (0, 1, 3, 6, and 12 h). To analyze the physical association of p62 puncta with autophagic vacuoles containing LC3, HeLa cells were left untreated or treated with 10 µM XIE62-1004 or XIE2008 compound for 16 h followed by immunostaining with anti-p62 and LC3 antibodies.

### In vitro oligomerization assay

HEK293 cells were transiently transfected with a plasmid DNA encoding p62-myc/his using Lipofectamine 2000. Approximately 24 h after transfection, cells were lysed with cell lysis buffer (50 mM HEPES, pH 7.4, 0.15 M KCl, 0.1% Nonidet P-40, 10% glycerol, protease inhibitors, and phosphatase inhibitors). Following a cycle of freeze/thaw, the cell suspension was incubated on ice for 1 h and centrifuged at 13,000 × *g* for 20 min at 4 °C. Protein concentration was determined using the Bradford assay. For the p62 oligomerization experiments, 0.5 µg protein was diluted in oligomerization assay buffer (16.7 mM HEPES, pH 7.4, 0.05 M KCl, 0.033% Nonidet P-40, 3.33% glycerol, protease inhibitors, and phosphatase inhibitors) and incubated with or without dipeptides (dissolved in water at a final concentration of 0.5 or 1 M) for 2–4 h in the presence of 100 μM bestatin at room temperature. Samples were mixed with a non-reducing loading buffer containing 4% lithium dodecyl sulfate (LDS), heated at 95 °C for 10 min, and resolved on 3% stacking and 12% separating SDS-PAGE. The monomer, oligomer, and aggregate forms of p62 were detected with a mixture of p62 and myc antibodies. To investigate the effect of XIE compounds on p62 aggregate formation, HEK293 whole cell extracts containing ectopically expressed p62 were incubated with XIE62-1004 at various concentrations (0, 10, 100, and 1000 μM) for 2 h at room temperature. For LC–MS/MS analysis, myc/His tagged p62 was overexpressed in HEK293 cells for 24 h. Two milligrams of total protein was subjected to R-nsP4 pulldown for the enrichment of p62. For 3 h, captured p62 was eluted with 50 mM RIFS tetrapeptides, and eluted p62 was simultaneously allowed to form oligomers. p62 monomer, oligomers and polymers were separated by nonreducing SDS-PAGE. Following Coomassie brilliant blue staining, bands representing p62 oligomers were excised and analyzed for LC–MS/MS.

### LC–MS/MS analysis

Gel pieces containing p62 oligomers were sliced and destained with 50% acetonitrile in 50 mM ammonium bicarbonate. The destained gel was alkylated with 55 mM IAA(Iodoacetamide) on free cysteine residues in dark environment. The MS grade trypsin protease (50 ng/μl) (Pierce Biotechnology, IL, USA) was added and followed by incubation at 37°C for 12 h. The resulting tryptic peptides were desalted using Pierce C-18 spin columns (Pierce Biotechnology, IL, USA) and dried using Speed-Vac (Scanvac; LaboGene Aps, Lynge, Denmark). The digested peptides were resuspended in mobile phase (0.1% formic acid in water) for LC–MS analysis. The HPLC analysis was performed on Easy-nLC 1000 system (Thermo Fisher Scientific Inc., Germany) equipped with a trap column (C18, 75 μm × 2 cm, 5 μm, Thermo Scientific Inc., Germany) for cleanup and an in-house analytical column (C18, 75 μm × 70 cm, 3 μm). The peptides were separated using the mobile phase comprising of solvent A (0.1% formic acid in water) and solvent B (0.1% formic acid in acetonitrile) in a gradient elution mode with a total run time of 100 min. The optimized linear gradient elution program was set as follows: (*T*_min_/% of solvent B): _0_/5, _10_/10, _80_/40, _82_/80, _90_/80, _92_/5, _100_/5 and the flow rate was 300 nl/min throughout the run time. For LC–MS analysis, Easy-nLC 1000 system (Thermo Fisher Scientific Inc., Germany) was coupled to Q-Exactive mass spectrometer (Thermo Fisher Scientific Inc., Germany). The typical operating source conditions for MS scan in positive ESI mode were optimized as follows: spray voltage, 2.3 kV; heated capillary temperature, 320°C; and nitrogen was used as damping gas. All the spectra were recorded under identical experimental conditions. The scan range was set from *m/z* 400 to 2000. Resolution of precursor ions was set at 70,000. The precursor ions matched on inclusion list were selected by orbitrap analyzer for subsequent MS/MS analysis. The 10 most abundant ions were fragmented by data-dependent mode at a resolution of 17,500 with the exclusion duration of 30 s and the isolation window was performed with 2.0*m/z*. The normalized collision energies used were set at NCE = 30.

### p62 filter trap assay

HeLa cells were left untreated or treated with 10 μM XIE62-1004, 2 μM MG132 or 25 μM HCQ for 16 h. The cells were washed twice with ice-cold PBS, pelleted by centrifugation for 5 min at 2356 × *g* at 4 °C and lysed on ice for 30 min in 100 μl lysis buffer (20 mM Tris-HCl, pH 7.5, 137 mM NaCl, 1 mM EGTA, 10% glycerol, 1% NP-40, and phosphatase and protease inhibitors). Lysates were centrifuged at 4 °C for 15 min at 19,000 × *g*. The insoluble fraction (pellet) was solubilized by sonication in lysis buffer containing 2% SDS. Protein concentration was determined using the BCA protein assay kit. Proteins (10 μg) were trapped by filtration through a pre-wetted 0.2 μm Trans-Blot nitrocellulose membrane adapted to a 96-well dot blot apparatus followed by washing twice with 200 μl 0.1% SDS and then blocked in TBS (100 mM Tris-HCl, pH 7.4, and 150 mM NaCl) containing 3% nonfat milk for 1 h. Due to the 0.2-μm pore size of this membrane, only aggregated proteins are retained, while soluble proteins pass through the pores of the membrane. To detect p62 aggregates, the membranes were probed with anti-p62 or anti-ubiquitinated protein antibodies. For in vitro p62 filter trap assay, myc/his tagged p62 was expressed using the in vitro TnT reticulocyte lysate system. Reticulocyte lysate (1 μl) diluted 1:100 in an assay buffer (50 mM HEPES, pH 7.4, 0.15 M KCl, 0.1% Nonidet P-40, 10% glycerol, and protease and phosphatase inhibitors) was incubated in the absence or presence of XIE62-1004 compound. To investigate the effect of XIE62-1004 on the autophagic clearance of mHTT (GFP-HDQ103), cell lysates from HeLa cells transfected with either wild-type huntingtin (GFP-HDQ25) or mHTT (GFP-HDQ103) were filtered through a pre-wetted 0.2-μm cellulose acetate membrane.

### Blue native polyacrylamide gel electrophoresis analysis

BN-PAGE was performed as described in Fiala et al.^69^. Briefly, MEFs were lysed with ice-cold BN-lysis buffer (20 mM Bis–Tris, pH 7.0, 500 mM ε-aminocaproic acid, 20 mM NaCl, 10% glycerol, 0.5% Triton X-100 and a mixture of proteases and phosphatase inhibitors). Cell lysates were placed in a dialysis membrane (molecular cut-off of 10 kDa) and dialyzed in BN-dialysis buffer (20 mM Bis–Tris, pH 7.0, 500 mM ε-aminocaproic acid, 20 mM NaCl, 10% glycerol, 0.1% Triton X-100 and a mixture of proteases and phosphatase inhibitors) overnight in the cold room. About 40 μg dialyzed proteins were separated in a 4–15% gradient blue native polyacrylamide gel in native condition. An excised BN-PAGE gel slice containing p62 molecules was placed on the top of a non-reducing SDS-polyacrylamide gel by rotating it 90 degrees in counterclockwise after boiled in 2× SDS sample buffer without β-mercaptoethanol. p62 species in the gel slice was separated by SDS-PAGE. To analyze the states of p62, immunoblotting analysis was performed using anti-p62 antibody.

### Cycloheximide-chase protein degradation assay

HeLa cells at 80% confluence were treated with 50 μg/ml cycloheximide following pre-incubation with the XIE2008 compound for 3 h. At indicated time points, cells were lysed on ice for 30 min in RIPA buffer containing a protease inhibitor cocktail, followed by centrifugation for 20 min at 15,000 × *g*. After centrifugation, 10 μg of total protein was subjected to immunoblotting.

### The enzyme-linked immunosorbent assay

Mouse embryonic fibroblasts (MEFs) lacking p62 were transfected with plasmids encoding full length p62 (p62-FL) or truncated p62 (p62-D3) using Lipofectamine 2000. After 24 h, cells were harvested and lysed in a lysis buffer (20 mM HEPES, pH 7.6, 0.15 M KCl, 0.1% NP-40, 10% glycerol, and protease inhibitors) followed by centrifugation at 15,392 × *g* for 20 min at 4 °C. Cell extract (20 μg) was incubated with GST-tagged LC3 recombinant protein that was immobilized on GSH-coated plates in the absence or presence of various concentrations of type-1 and type-2 dipeptides or XIE62-1004 for 1.5 h at room temperature. Bound p62 was detected by incubation with anti-p62 antibody for 1 h at room temperature followed by incubation with HRP-conjugated secondary antibody for 45 min at room temperature. After washing three times with PBS, TBM substrate was added to each well, and color was developed in the dark at room temperature for 10 min. TMB stop solution, 2 N H_2_SO_4_, was added to stop the color reaction. Observance was measured on a plate reader at 450 nm.

### RNA interference analysis

Cells in a 12-well plate (0.5 × 10^6^ per well) were transfected with 40 nM siRNA using RNAi Max reagent. Pre-designed Silencer Select siRNAs (Invitrogen) knockdown SQSTM1 using the following sequences: siSQSTM1 (HSS113117-sense, AUAGUUCUUGGUCUGCAGGAGCCUG; HSS113117-antisense, CAGGCUCCUGCAGACCAAGAACUAU).

### Statistical analysis

Data are presented as mean ± S.D. of three independent experiments. Statistical analysis was performed with Prism 6 software (Graph Pad) using ANOVA. Differences with *P* < 0.05 were considered statistically significant (****P < 0.0001; ***P < 0.001; **P < 0.01; *P < 0.05; n.s., no statistically significant difference (*P* ≥ 0.05).

### Data availability

All data supporting the findings of this study are available within the article and its supplementary information files or from the corresponding author on reasonable request.

## Electronic supplementary material

Supplementary Information
