# Supplementary material for: p62/SQSTM1/Sequestosome-1 is an N-recognin of the N-end rule pathway which modulates autophagosome biogenesis
Source: Nat Commun. 2017 Jul 24;8:102. doi: 10.1038/s41467-017-00085-7 (PMC5524641; doi:10.1038/s41467-017-00085-7)
Supplement: Supplementary file 1 — Supplementary Information [file 41467_2017_85_MOESM1_ESM.pdf]

Title of file for HTML: Supplementary Information
Description: Supplementary Figures

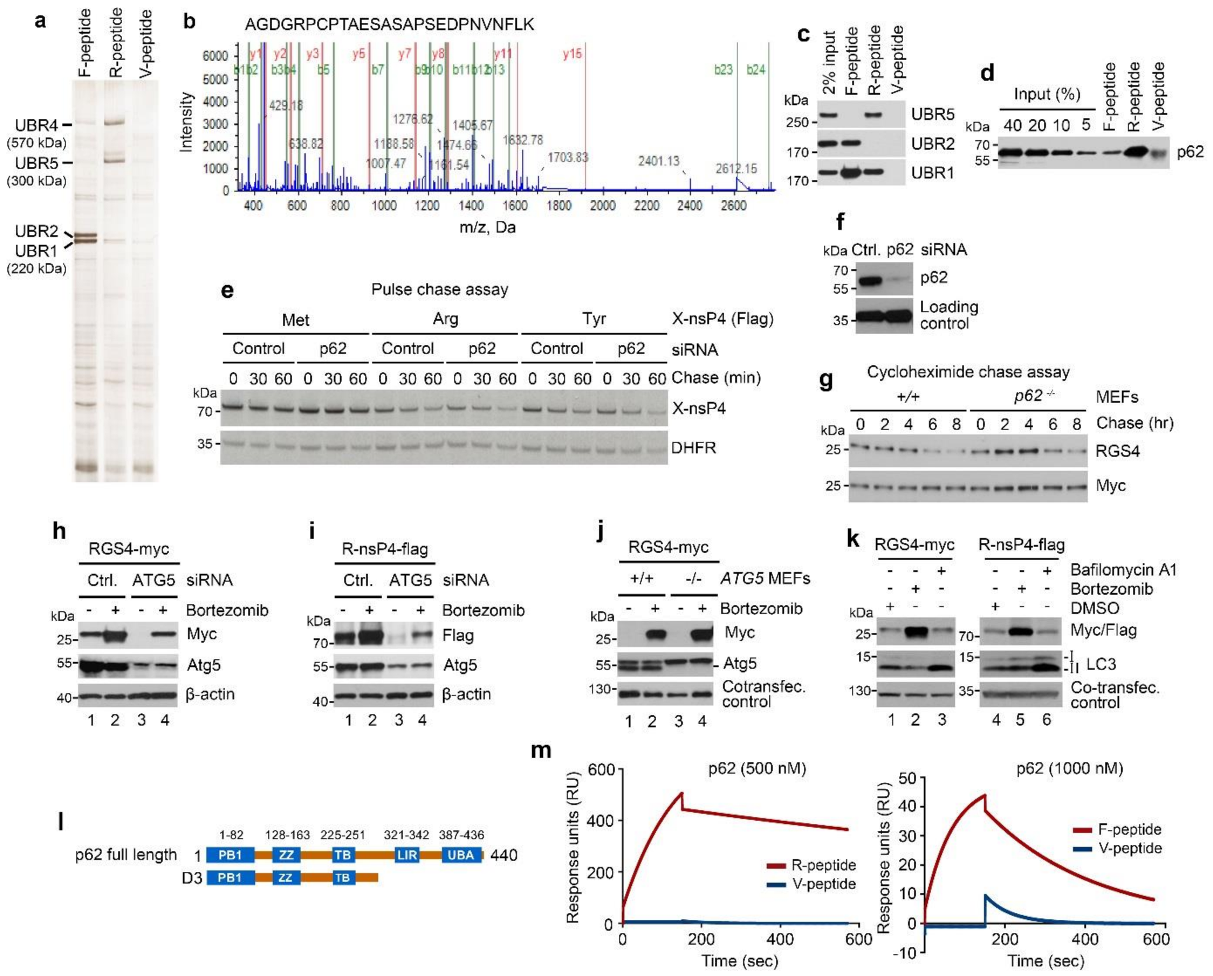

Supplementary Figure 1. Identification of p62 as an N-recognin of the autophagic N-end rule pathway. (**a**) *In vitro* peptide pulldown assay using testis extracts and bead-conjugated peptides. (**b**) Mass spectrometric analysis of p62 peptide. (**c**) Western blot analysis of proteins partially purified as described in **a**. (**d**) p62 was expressed using the TnT system, followed by X-peptide pulldown assay. (**e**) Pulse chase assay using X-nsP4. X-nsP4 and DHFR (control) were detected by immunoblotting with anti-flag antibody. (**f**) Knock down efficiency of p62 RNAi. (**g**) Cycloheximide chase assay using RGS4. (**h**) Western blot analysis was performed to assess the importance of the UPS and autophagy in RGS4 degradation using bortezomib (10 μM) as a proteasome inhibitor and *ATG5* RNAi to impair autophagy. (**i**) HEK293 cells expressing R-nsP4-flag was used in western blot analysis as described in **h.** (**j**) MEFs expressing RGS4-myc was used in western blot analysis as described in **h.** (**k**) HEK293 cells expressing RGS4-myc and R-nsP4 were treated with either DMSO, 10 μM bortezomib, or 200 nM bafilomycin A1 for 4 h (**m**) Schematic representation of C-terminal deletion mutants of p62-D3 construct. (**n**) Biacore sensorgrams showing comparison of Arg-peptide versus Val-peptide and Phe-peptide versus Val-peptide.

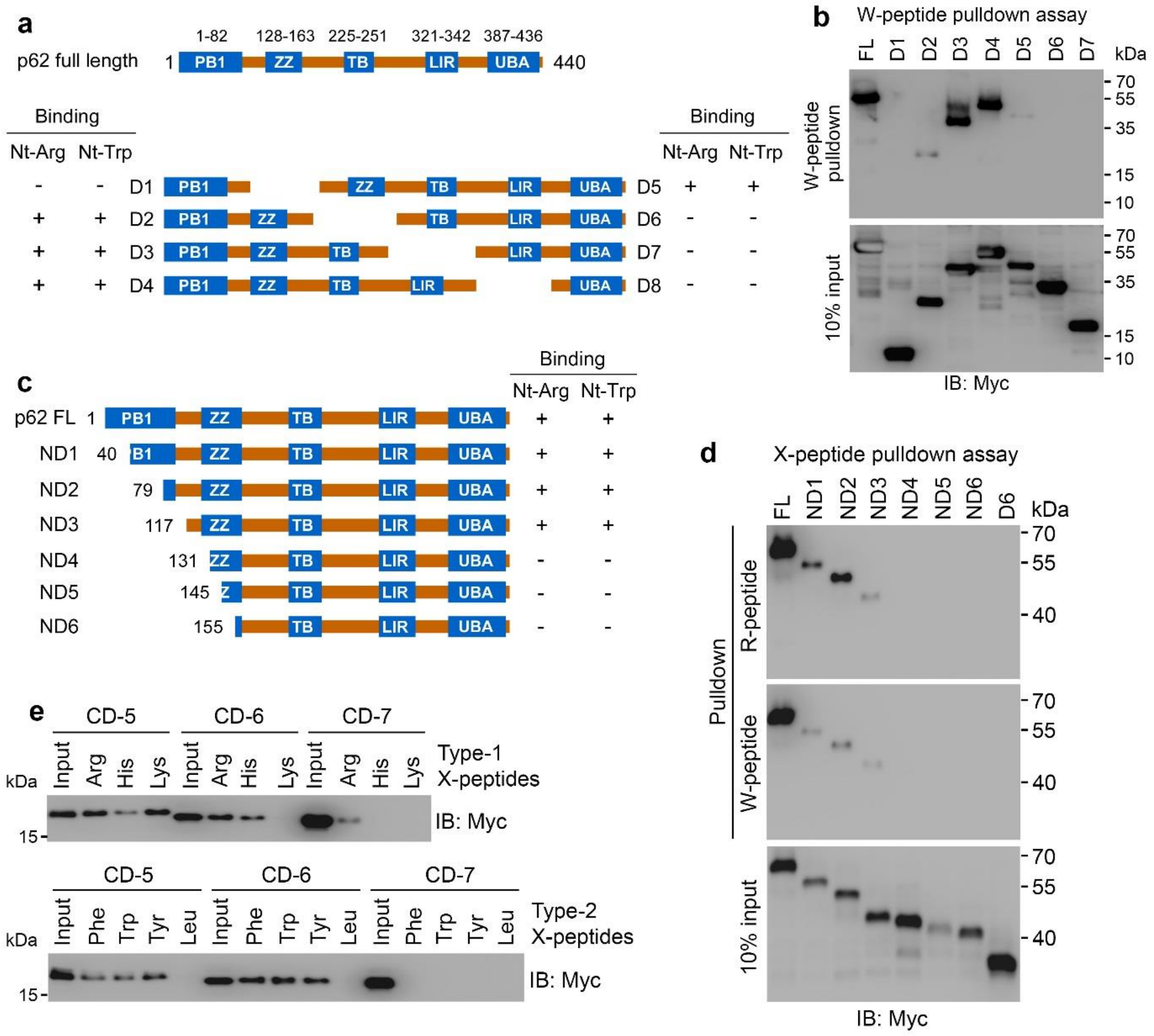

Supplementary Figure 2. The ZZ domain of p62 is a ligand binding domain for N-end rule N-terminal amino acids. (**a**) Schematic representation of p62 deletion mutants. (**b**) Trp-peptide pulldown assay using constructs presented in **a**. (**c**) Schematic representation of N-terminal deletion constructs. (**d**) X-peptide pulldown assay using constructs presented in **c**. (**e**) p62 D3 (#1-310) was C-terminally deleted to generate CD1 through CD9. Amongst these, CD5, CD6, and CD7 (see Fig. 1e) were ectopically expressed in HEK293 cells and subjected to X-peptide pulldown assay (X= Arg, His, Lys, Phe, Trp, Tyr, and Leu).

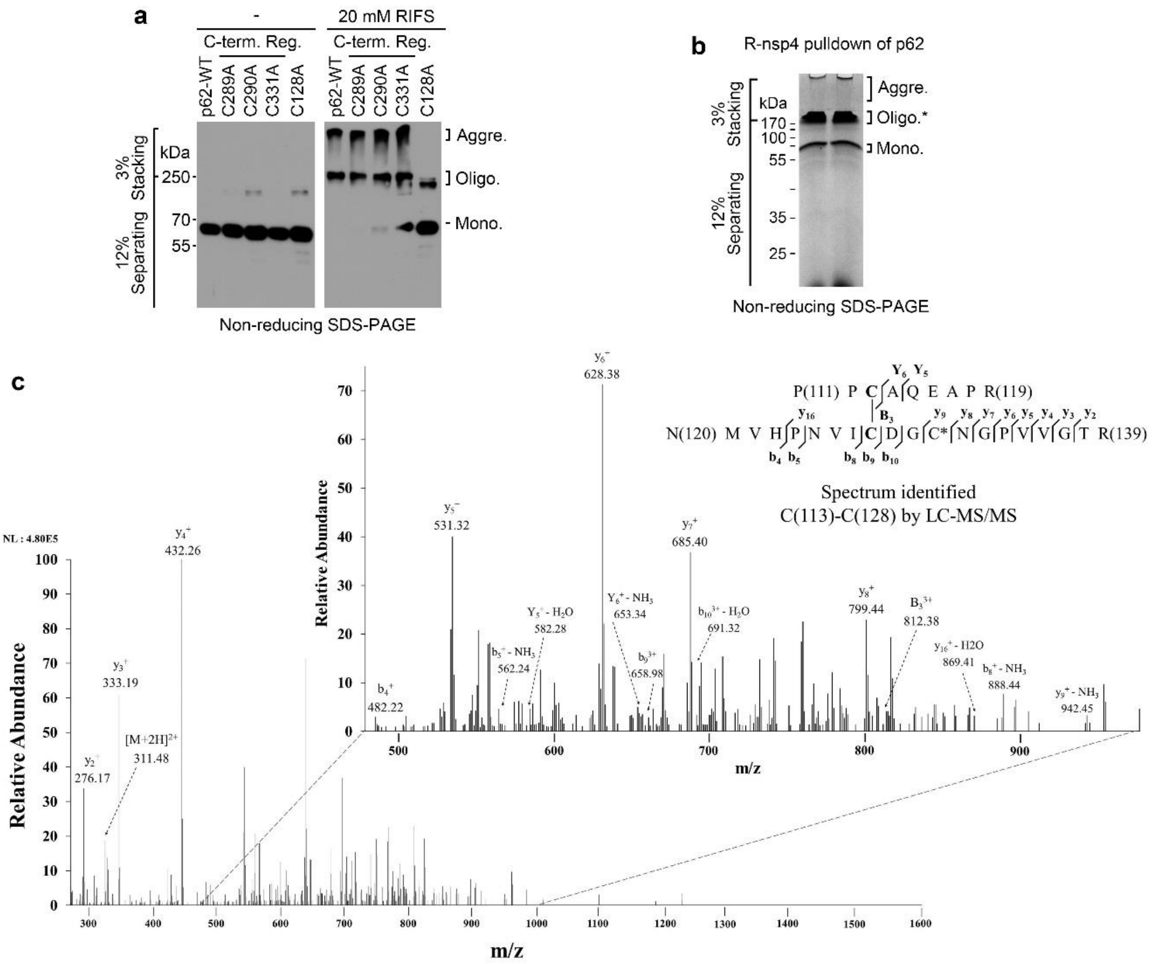

Supplementary Figure 3. A single C/A nucleotide substitution in the C-terminal region of p62 showed no effect on Nt-Arg induced p62 polymerization. (**a**) *In vitro* p62 oligomerization using RIFS tetra peptides and C-terminal C/A mutants. Cell extracts containing myc/His tagged p62 wild type and mutants were incubated with or without 20 mM RIFS tetrapeptides for 1.5 h at RT. Forms of p62 was detected employing immunoblotting analysis. (**b**) Enrichment of p62 using R-nsP4 pulldown. Myc/His tagged p62 was overexpressed in HEK293 cells. 2 mg total protein was used for R-nsP4 pulldown assay. Bound p62 was eluted with 50 mM RIFS tetrapeptides, and simultaneously, eluted p62 was allowed to form oligomers. Following Coomassie staining, bands representing p62 oligomers were excised for LC-MS/MS. (**c**) LC-MS/MS analysis of p62 oligomers. p62 oligomers were in-gel alkylated with 55 mM IAA (Iodoacetamide) on free cysteine residues followed by tryptic digestion before LC-MS/MS analysis.

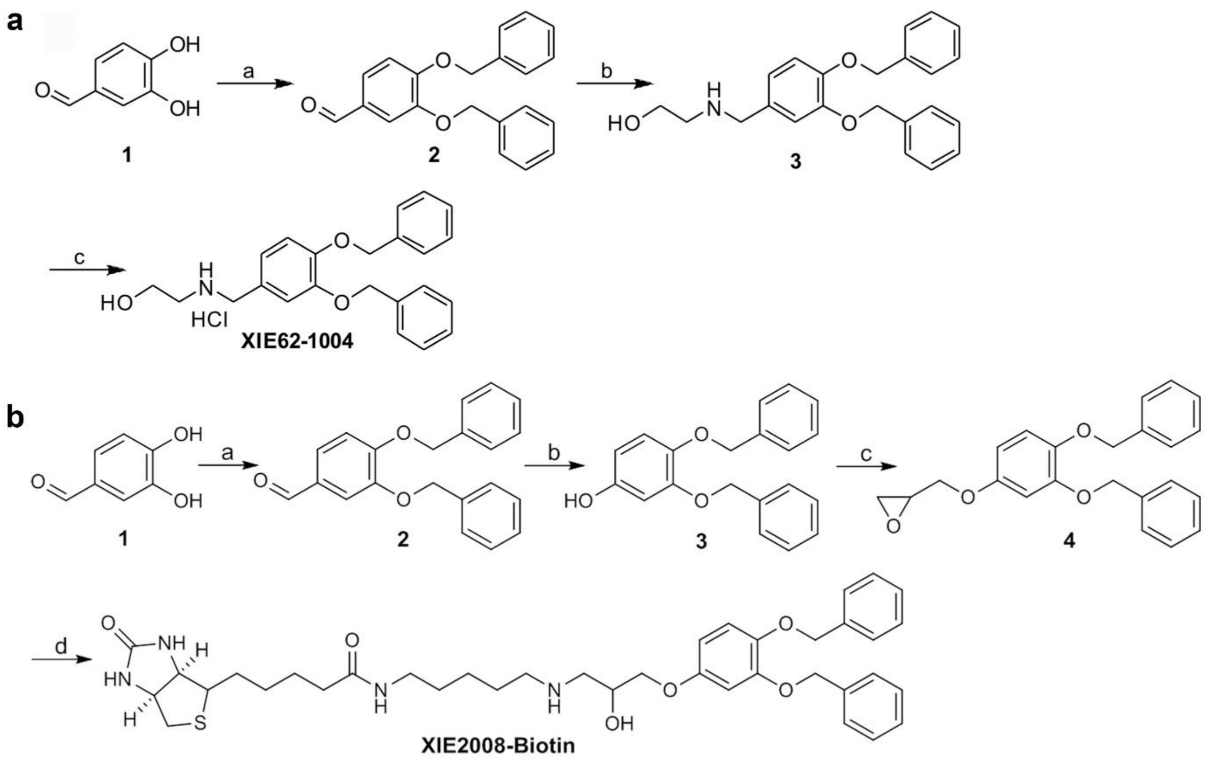

Supplementary Figure 4. Synthesis of small molecule ligands to p62 ZZ domain. (**a**) ***Synthesis of XIE62-1004. Reagents and conditions:*** (a) (bromomethyl)benzene, K_2CO_3, DMF, 65 ^{o}C, 12 h; (b) (i) 2-aminoethan-1-ol, EtOH, overnight, 60 °C; (ii) $NaBH_4$, MeOH, r.t, 12 h; (c) HCl (gas), EtOH, r.t, 1 h. (**b**) ***Synthesis of biotinylated XIE2008. Reagents and conditions:*** (a) BnBr, NaH, THF, 2h; (b) (i) mCBPA, DCM, 6h; (ii) 6N NaOH, MeOH; (c) epichlorohydrin, 4N KOH, MeOH, 2h; (d) EZ-link Pentylamine biotin, MeOH, reflux, 2h.

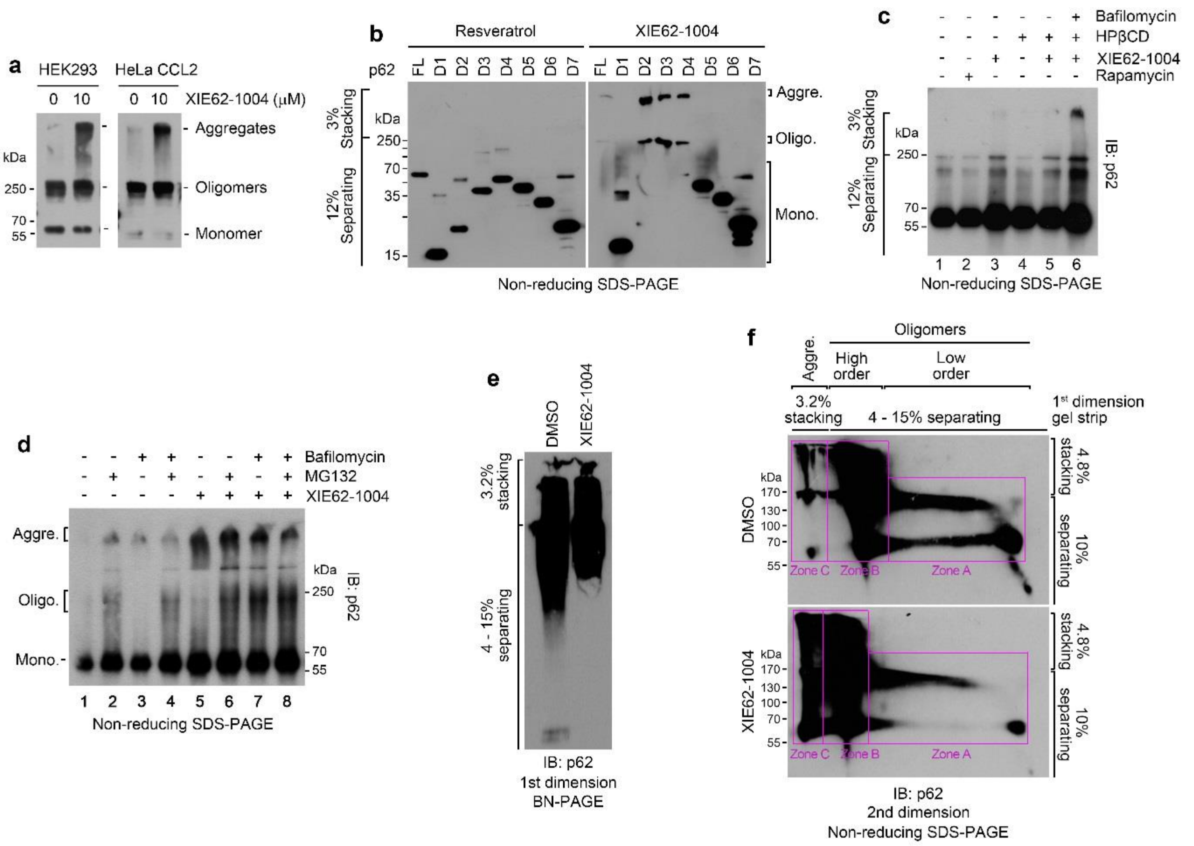

Supplementary Figure 5. XIE62-1004 and XIE2008 induce p62 polymerization. (**a**) *In vitro* p62 oligomerization assay using XIE62-1004. HEK293 and HeLa cell extracts containing overexpressed p62 with myc/His tag were incubated with XIE62-1004 for 2 h at RT. Forms of p62 were detected by immunoblotting analysis after separated by non-reducing SDS-PAGE. (**b**) *In vitro* p62 oligomerization assay using XIE62-1004 and cell extracts containing p62 deletion mutants. Forms of p62 were detected as described in a. (**c**) *In vivo* p62 oligomerization assay. HeLa cells were treated with 5 μM XIE62-1004 for 24 h, 200 nM rapamycin, 1 mM HPβCD for 24 h and 200 nM bafilimycin for 18 h followed by soluble and insoluble fractionation. The insoluble fractions were separated by non-reducing SDS-PAGE. (**d**) *In vivo* p62 oligomerization assay using XIE62-1004 as well as MG132 and bafilomycin. HeLa cells were treated with 5 μM XIE62-1004 for 24h, 10 μM MG132 for 18 h and 200 nM bafilomycin for 18h. Following soluble and insoluble fractionation, forms of p62 in the insoluble fractions were visualized as described in a. (**e**) MEF cell lysates were treated with either DMSO or 1 mM XIE62-1004 for 2 h at room temperature and subjected to western blot analysis using anti-p62 antibody. (**f**) An excised BN-PAGE gel slice containing p62 molecules was placed on the top of a non-reducing SDS-polyacrylamide gel by rotating it 90 degrees in counterclockwise. p62 species in the gel slice was separated by SDS-PAGE, and the states of p62 were analyzed by immunoblotting using anti-p62 antibody.

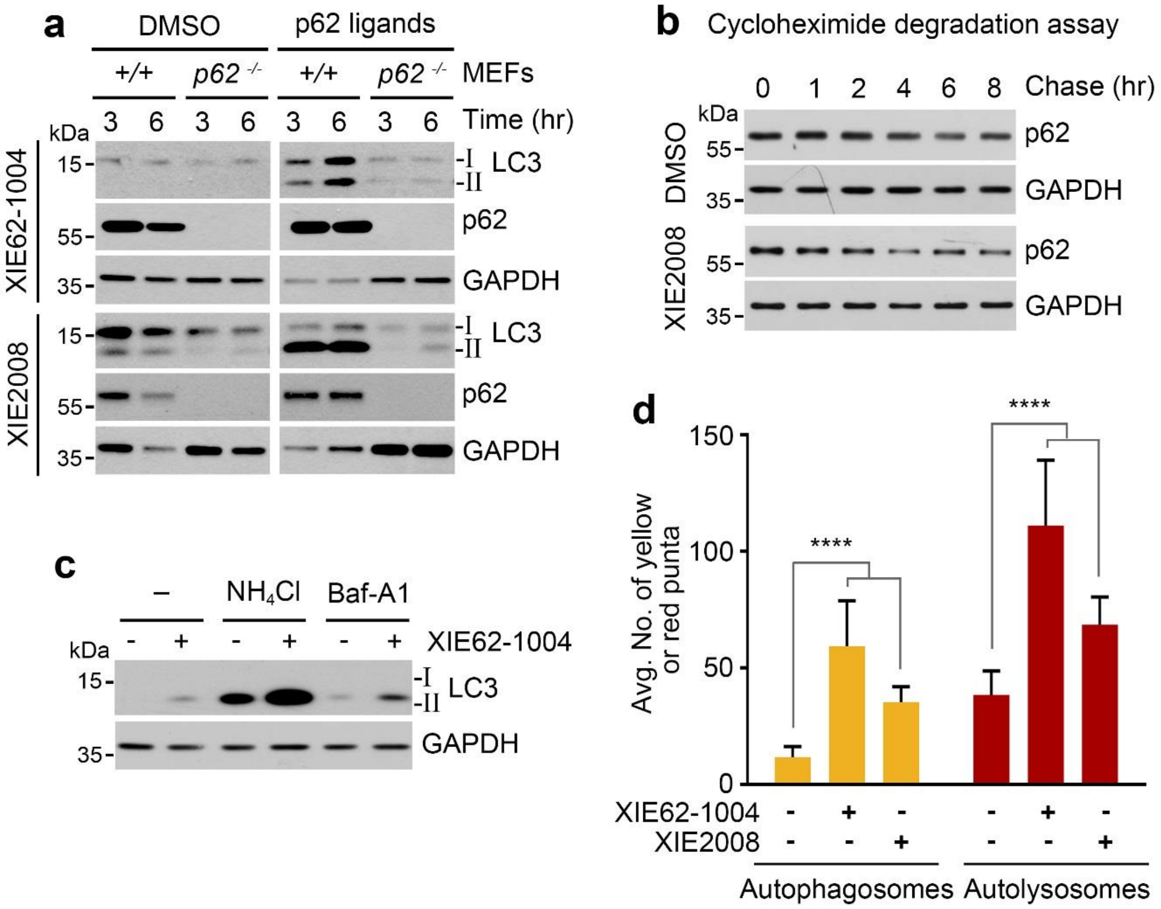

Supplementary Figure 6. XIE62-1004 and XIE2008 induce autophagy by stimulating autophagosome formation. (**a**) Autophagosome formation analysis showing that XIE62-1004 and XIE2008 fail to induce autophagy in *p62*$^{-/-}$ MEFs. (**b**) Cycloheximide degradation assay of p62. HeLa cells were pre-treated with either DMSO or 10 µM XIE2008 for 3 h followed by cycloheximide chase using 20 µg/ml cycloheximide. At indicated time points, cells were lysed, and p62 was detected using immunoblotting analysis with anti-p62 antibody. (**c**) Autophagic flux assay. HeLa cells were treated with 25 mM NH_4Cl or 0.1µM bafilomycin for 3 h in combination with 10 µM XIE62-1004 for 6 h, followed by immunoblotting analysis. Note that ZZ ligands and autophagy blockers show synergistic effects in inducing autophagosome formation. (**d**) Quantification of Fig. 5f. The results represent the average puncta number of 40 cells pooled from two independent experiments. Statistical significance was calculated using a one-way ANOVA test (**** $p \leq 0.0001$).

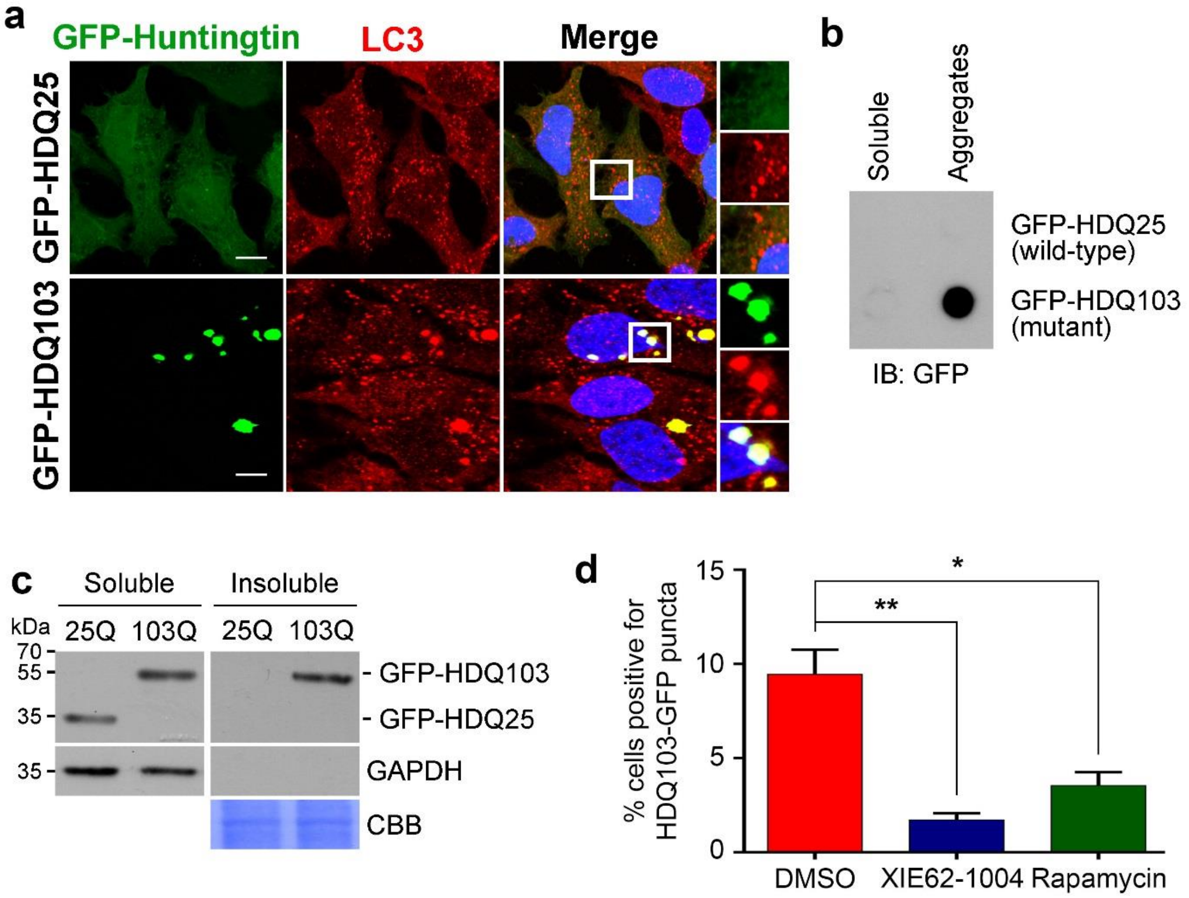

Supplementary Figure 7. XIE62-1004 promotes the removal of mHTT. (a) Immunocytochemistry of LC3 in HeLa cells expressing either GFP-HD-Q25 or GFP-HD-Q103. (**b**) Filter trap assay of GFP-HD-Q25 and GFP-HD-Q103. (**c**) GFP-HD-Q25 and GFP-HD-Q103 transiently expressed in HeLa cells were fractionated into soluble and insoluble proteins in 1% Triton X100, followed by immunoblotting analysis. (**d**) Quantitation of Fig. 6b. Shown are the percentages of cells positive for GFP-HD-Q103 (n = 3). A one-way ANOVA test was performed to determine statistical significance (* $p \leq 0.05$; ** $p < 0.01$).

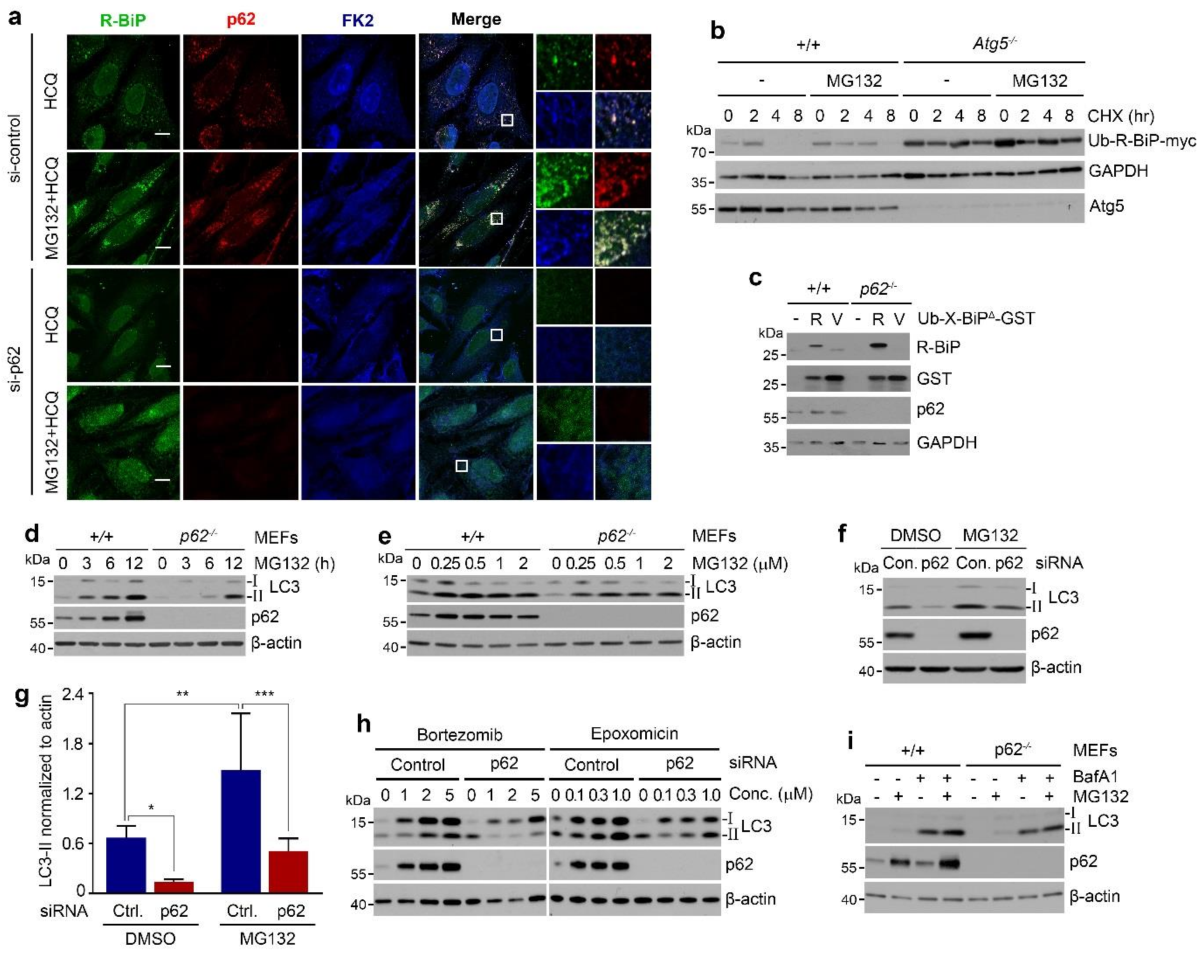

Supplementary Figure 8. p62 is required to induce autophagy in response to proteasomal inhibition. (**a**) Immunofluorescence analysis of R-BiP, p62 and Ub-cargoes. HeLa cells were transfected with either control or p62 siRNA and incubated with 25 μM hydroxychloroquine (HCQ) in the absence or presence of MG132 for 18 h. Scale bar, 10 μm. (**b**) Degradation of R-BiP by both proteasomes and lysosomes. Ub-R- BiP-myc/His was ectopically expressed in wild type and Atg5$^{-/-}$ MEFs (**c**) p62 dependent turnover of R-BiP$^{\Delta}$-GST. The wild type and knock out p62 MEFs were transiently transfected with Ub-X- BiP$^{\Delta}$-GST for 30 h. (**d**) Wild-type and *p62*$^{-/-}$ MEFs with 1 μM MG132, followed by time-course immunoblotting. (**e**) Wild-type and *p62*$^{-/-}$ MEFs with MG132 at concentrations as indicated for 6 h. **(f)** Analysis of LC3-I to LC3-II conversion using control and p62 knocked down HEK293 cells with 1 μM MG132. **(g)** Quantification of **f**. The intensity of LC3-II band was normalized to β-actin. Presented are averages of three independent experiments. Statistical significance was calculated using a two-way ANOVA test (* $p < 0.05$; ** $p < 0.01$; *** $p < 0.001$). (**h**) HEK293 cells were transfected with control or p62 siRNA and incubated with proteasome inhibitors, bortezomib and epoxomicin, at concentrations indicated in the figure for 16 h. (**i**) Analysis of autophagosome biogenesis using p62 wild type and knockout MEFs.

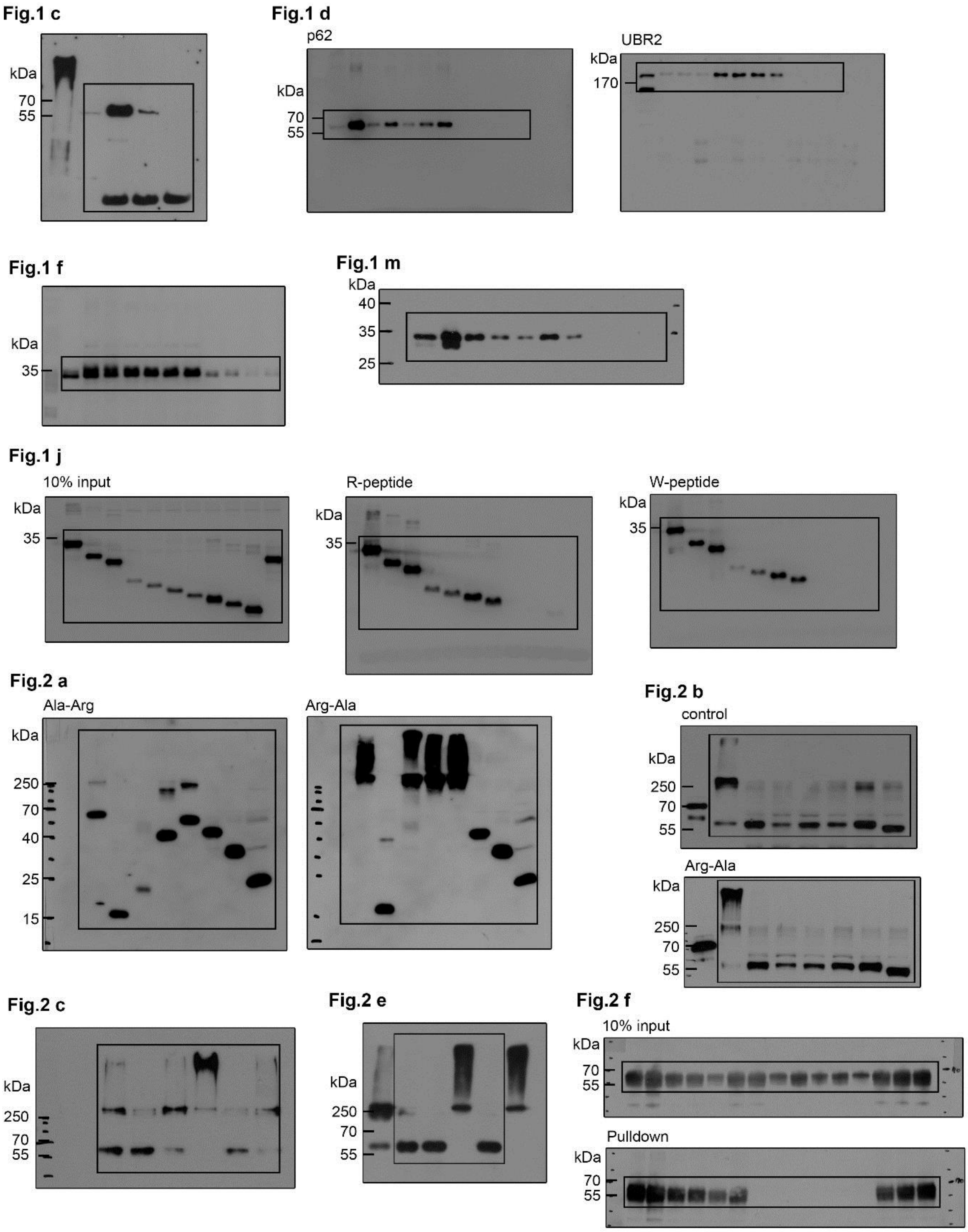

Supplementary Figure 9. Scanned original immunoblots

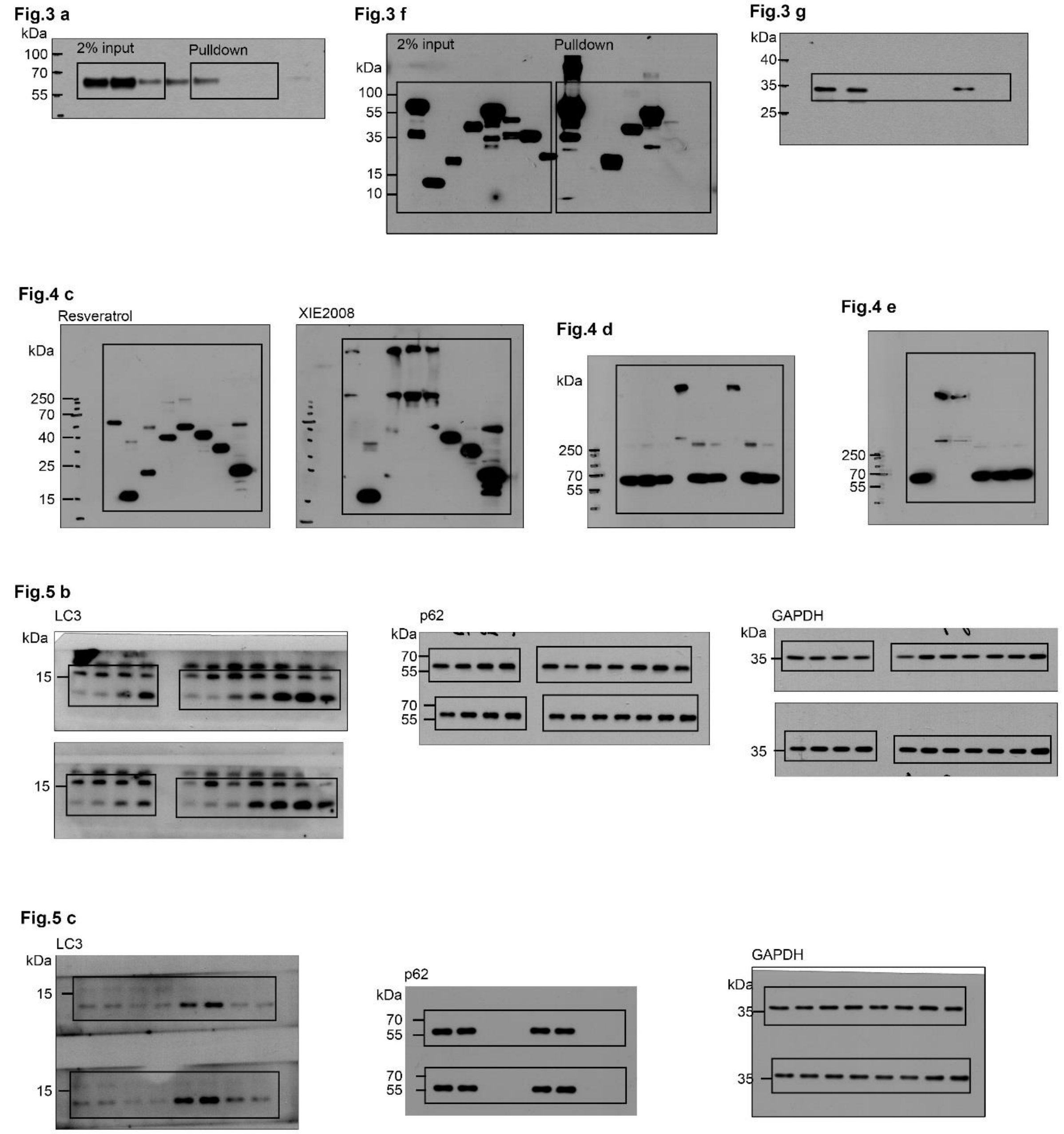

Supplementary Figure 9. (continued)

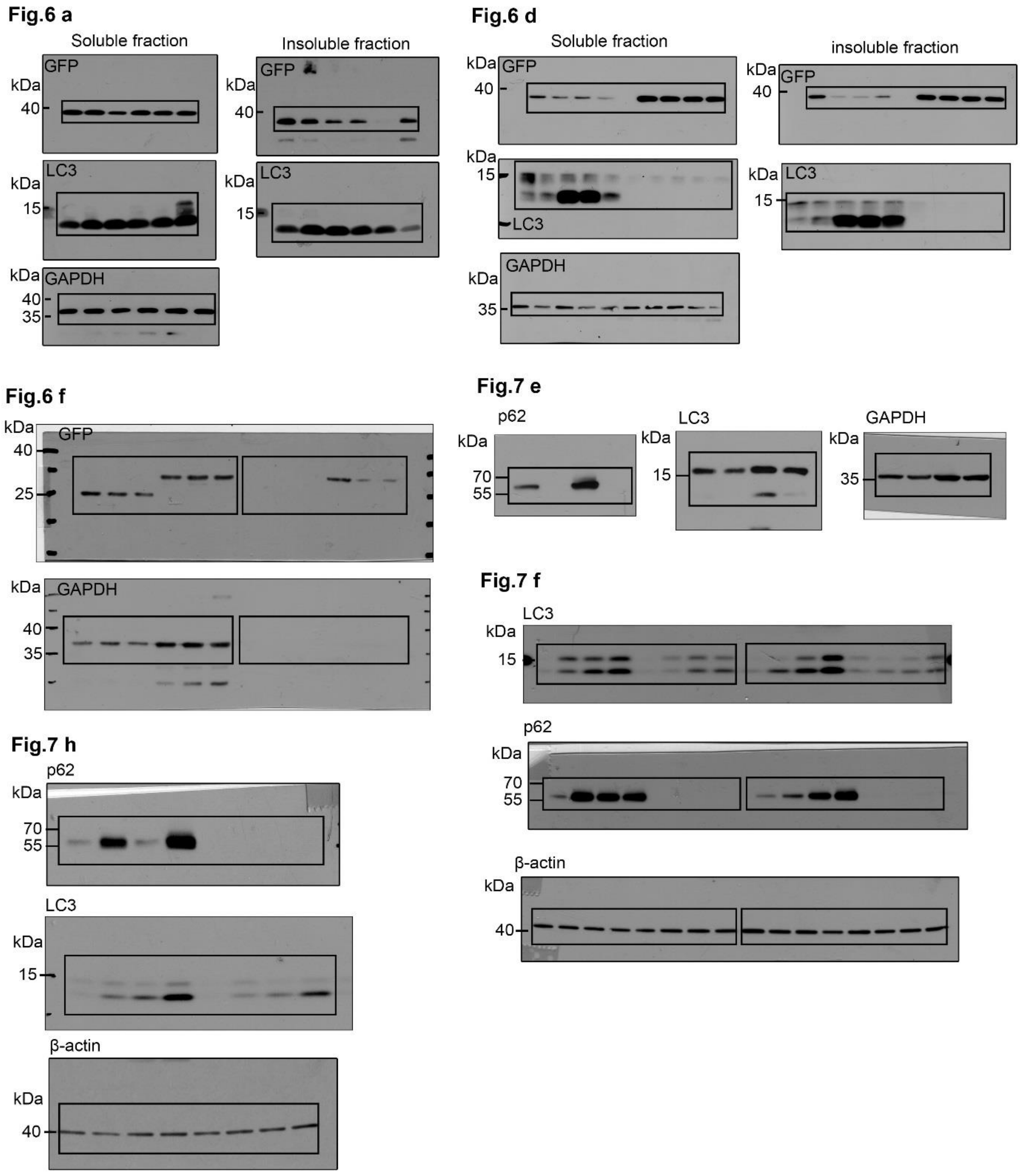

Supplementary Figure 9. (continued)
